# Synthesis and Characterization of Rubisco–Magnesium Complexes for Potential Gas Adsorption Applications

**DOI:** 10.1021/acsomega.5c08077

**Published:** 2026-01-08

**Authors:** Gia Huy Pham, Elizabeth Willenborg, Emily Weber, Brandon Robinson, Cerasela Zoica Dinu

**Affiliations:** Department of Chemical and Biomedical Engineering, 5631 West Virginia University, Morgantown, West Virginia 26506, United States

## Abstract

Enzyme-based complexes represent an emerging class of functional adsorbents combining specificity and environmentally friendly potential. We proposed the development of metal-enzyme-based complexes that leverage the unique properties of magnesium metal to increase enzyme-structure integration for the formation of hybrid porous matrices with the potential to modulate targeted gas adsorption under mild conditions. For this, ribulose-1,5-bisphosphate carboxylase oxygenase (RuBisCO), a carboxyl-lyase responsible for carbon fixation in the Calvin–Benson–Bassham cycle, was used as a scaffold that supported stable coordination of its amino acids, water, and/or phosphate groups with magnesium ions. Time- and dose-dependent synthesis and characterization of the resulting metal–enzyme complexes, performed through electron microscopy, infrared spectroscopy, and X-ray diffraction, unraveled the high-resolution structure formation. Magnesium integration led to crystal lattice formation, resulting in complexes of defined porosity and size, as evaluated through particle diffraction studies helping connect metal–enzyme synthesis time and ratio with the observed physicochemical properties. Preliminary gas adsorption testing with N_2_ and CO_2_ conducted using both physisorption and static chemisorption methods demonstrated that the metal–enzyme complexes exhibit measurable gas uptake behavior, thus indicating potential for gas interaction and adsorption under controlled conditions. These findings lay the groundwork for further exploration of metal–enzyme hybrids as tunable, bio-inspired materials for gas adsorption, with future studies needed to optimize performance and assess such complexes potential in real-world applications.

## Graphical abstract

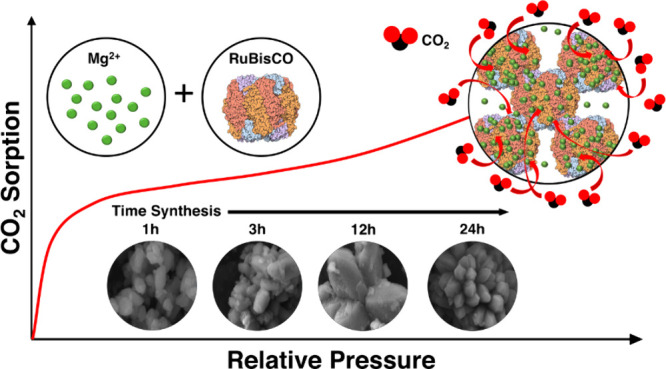

## 1 Introduction

Global, industrial, and societal needs led to scientific efforts focused on the development of carbon capture and sequestration (CCS) technologies^1–3^ with the goal of decreasing CO_2_ emissions. Studies focused on process design and optimization at pre- ^4–6^ and postcombustion^4,7,8^ and oxy-fuel combustion,^9,10^ with analysis showing that CCS performed at the postcombustion stage for instance is a more economically viable option for commercial scale and established industrial infrastructure implementation.^11,12^ Complementarily, other CCS studies focused on development of absorption-based porous material^13,14^ with CO_2_ (1) physically bound through weak van der Waals forces (i.e., to activated carbons^15,16^ and mesoporous silica^17,18^ ), (2) chemically bound through covalent interactions (i.e., to metal–organic frameworks (MOFs) such as MUF-16,^19,20^ BUCT-C19,^21^ and Mg_2_(dobpdc) (dobpdc^4‑^ = 4,4'-dioxidobiphenyl-3,3'-dicarboxylate)^22,23^ ) or, in the case of zeolitic imidazolate framework-8 (ZIF-8), through both physical^24,25^ and chemical-binding behavior,^26,27^ respectively. Upon adsorption and conjugation with amine-based sorbents like monoethanolamine (MEA)^28,29^ or with solvents like polyethylene glycol (PEG) in the Rectisol process^30,31^ and propylene carbonate in the FLUOR process,^32,33^ CO_2_ molecules were transformed into less harmful carbon-based products.^13,34,35^

While these technologies give a wide range of selections to capture and/or dissolve/transform CO_2_ molecules, each faces unique drawbacks and challenges that preclude further upscaling and optimization for long-term commercial implementation and usage. Briefly, degradation at elevated temperature was for instance observed for MEA at 135 °C with 2.5–6% degradation/week^36^ and degradation rate of 57.6% after 5 weeks.^37^ Complementarily, instability was recorded for Mg_2_(dobpdc) above 25 °C and at >50% relative humidity.^38^ Lack of stability upon repeated cycles of adsorption with 50–90% loss in CO_2_ uptake was observed for amine-functionalized adsorbents on silica support, with multiple temperature swing adsorption cycles and long-time CO_2_/air exposure at 150 °C;^39^ complementarily, 55% loss in CO_2_ capture capacity and cycling stability was observed for Ca_0.9_Mg_0.3_O.^40^ Furthermore, it was also shown that the reported CCS technologies require high energy (either thermal,^41–44^ electrical,^45–47^ or chemical^48–50^ ) for materials regeneration via CO_2_ desorption, especially upon nonspecific, competitive binding of other gaseous molecules in ambient air or flue gas,^51–53^ or in membranes.^54–56^

Recently, enzymes-based technologies integration was proposed^57–61^ as a means to increase adsorption and CO_2_ binding for increased gas capturing,^58,60,62^ reduce the energy cost for materials regeneration,^58,60,63–65^ increase stability with cyclic uptake,^58,62,65,66^ and capture and quickly convert the gas, respectively.^67^ Indeed, carbonic anhydrase (CA),^60–62,68^ a zinc­(II) (Zn^2+^)-containing metalloenzyme with high levels of catalytic activity of hydrolyzing CO_2_ at a maximum rate of 10^6^ s^–1^,^69–71^ was shown to exhibit the greatest binding affinity^72^ with a tetrahedral Zn­(II) center,^73,74^ immediately followed by an octahedral Co­(II) center.^72,75^ Complementarily, formate dehydrogenase (FDH),^76–79^ a molybdenum (Mo)- or tungsten (W)-containing metalloenzyme^80–82^ that naturally catalyzes the redox reaction of CO_2_ to formate (HCOO^–^) using reduced cofactors such as nicotinamide adenine dinucleotide (NADH) or nicotinamide adenine dinucleotide phosphate (NADPH),^83–85^ was shown to possess enhanced activity and stability upon immobilization onto mesoporous silica (MPS),^76,86,87^ graphite electrodes,^88^ zeolites,^89^ and ZIFs.^90^ However, despite the increased CO_2_ specificity of adsorption, binding, and ultimately transformation,^67,91^ optimization challenges as related to enzyme operational stability^92–95^ and limited substrate uptake^96–98^ still hinder such infrastructure implementation in CCS technologies.^60,67,70,91–100^ Moreover, such enzymes still require either a humid atmosphere or cofactors for the CO_2_ adsorption and transformation.^67,101^

To increase the performance of enzyme-based integration for CO_2_ adsorption as the first step toward CCS technologies based on enzymatic systems, we propose the development of hybrid systems in which ribulose-1,5-bisphosphate carboxylase oxygenase (RuBisCO;^102–104^ EC 4.1.1.39),^105^ a carboxyl-lyase^106^ responsible for carbon fixation in the Calvin–Benson–Bassham (CBB) cycle,^103^ is used as a scaffold for magnesium coordination.^107,108^ Upon binding, Mg^2+^ coordination was hypothesized to drive enzyme-based complexes’ crystal lattice formation^109,110^ to result in structures with well-defined porosities due to its known ability to form stable interactions with amino acids,^111–115^ water,^116,117^ and phosphate groups.^111,117–119^ The development of metal–enzyme-based complexes was herein examined in the context of complex formation through one-pot time and dose-dependent reactions, with the synthesis and characterization providing insights into how such hybrid systems can unlock the potential of enzyme-based technology integration for a more sustainable approach to absorb CO_2_.

## 2 Experimental Section

### 2.1 Materials

Magnesium chloride hexahydrate 99% (MgCl_2_·6H_2_O, purchased from Acros Organics, USA) and d-ribulose-1,5-diphosphate carboxylase from spinach (RuBisCO, partially purified powder, 0.01–0.1 unit/mg solid, Sigma-Aldrich, USA) were used for the synthesis of the magnesium/ribulose-1,5-bisphosphate carboxylase oxidase (Mg:RuBisCO) complexes. Potassium phosphate buffered saline (K-PBS, 100 mM) buffer at pH 7.8 served as a solvent. The K-PBS buffer was prepared by combining ratios of monobasic potassium phosphate (Reagent ACS, crystals, Acros Organics, USA) and dibasic potassium phosphate (anhydrous crystalline powder, Fisher Chemical, USA) in DI water. Ultrahigh-purity nitrogen (N_2_, UHP grade, Matheson, USA) was used for N_2_ physisorption testing. Research-grade carbon dioxide (99.999%, CO_2_, Airgas, USA) was used for CO_2_ physisorption testing; 99.995% ultrahigh-purity CO_2_ (CO_2_ UHP grade, Matheson, USA) was used for CO_2_ static chemisorption testing, and ethylene glycol (technical grade, purchased from Thermo Scientific, USA) was used to cool or heat the temperature-controlled dewar during CO_2_ physisorption testing.

### 2.2 Synthesis of Metal–Enzyme-Based Complexes

Mg:RuBisCO complexes were formed via a room-temperature synthesis technique. Briefly, different ratios of MgCl_2_ salt were mixed with different ratios of purified RuBisCO enzyme (i.e., testing conditions included 5:1, 50:1, and 100:1 (mg:mg), respectively) in a glass vial containing 1 mL of K-PBS, pH 7.8. The resulting mixture was vortexed lightly followed by 10 s sonication to fully disperse the solid powders; subsequently, the mixture was stirred at 600 rpm for different time points associated with the synthesis (i.e., 1, 3, 6, 12, and 24 h, respectively). When time elapsed, the mixture was placed in a Falcon tube and centrifuged at 5000 rpm for 5 min to separate any formed product. Three consecutive washing steps (each with 10 times the initial volume used for the reaction) were used to remove any unreacted species. After the third wash, the precipitate was left in a Falcon tube with a vented wrap and dried in a vacuum desiccator for 24 h. After drying, the product yield values were recorded, with product subsequently being stored at 10 °C in a glass vial.

### 2.3 Characterization of the Metal–Enzyme Complexes

Synthesized complexes were evaluated by using a variety of techniques. Specifically, for morphologies analysis, complexes obtained at different ratios and times of synthesis were investigated by field emission scanning electron microscopy (FE-SEM) performed on a JEOL JSM 7600F SEM (JEOL, Massachusetts, USA) at 15 kV. For this, Mg:RuBisCO complexes were placed on double-sided carbon conductive tape on a SEM specimen stub and then sputtered with gold/palladium (Au/Pd) using a Denton Desk V Sputter and Carbon Coater (Denton Vacuum, New Jersey, USA) at 8 mA for 100 s. Concurrently, energy-dispersive X-ray spectroscopy (EDX) mapping was used to evaluate the elemental composition of the complexes. SEM and EDX collections were repeated at least 3 times for each ratio of synthesis and its associated time, respectively.

Chemical compositions of the synthesized Mg:RuBisCO complexes were evaluated by using the Digilab FTS 700/UMA 600 attenuated total reflectance Fourier transform infrared (ATR FTIR, Biorad Laboratories, California, USA) spectrometer. Scans were collected in the range 400–4000 cm^–1^ at a resolution of 4 cm^–1^. A total of 128 scans were coadded to form the final spectrum of each sample being investigated. FTIR collection was repeated at least 3 times for each ratio and its corresponding synthesis time, respectively.

Particle size analysis at different ratios and different time points of synthesis was carried out by a SALD-2300 laser-diffraction particle size analyzer (Shimadzu, Colombia, Maryland, USA) equipped with a red semiconductor laser at 680 nm. Briefly, 1 mg of sample was dispersed in 1 mL of K-PBS pH 7.8 with slight sonication. This mixture was then placed in a quartz glass cell containing approximately 10 mL of K-PBS pH 7.8 and analyzed. To negate the settling of particles to the bottom of the cell, a stirring plate was used to perform a slow vertical stirring movement. Particle size analysis was repeated 15 times for all ratios with the composites’ corresponding synthesis times, respectively. The means, medians, modes, and standard deviations of samples from each run were automatically calculated by the instrument’s proprietary program and averaged to obtain the standard deviation. The kurtosis and Pearson second coefficients of skewness were calculated using eq 1 and eq 2, respectively.

1 $$\mathrm{kurtosis}=\frac{1}{N}\overset{n}{\underset{i=1}{\sum }}{\left(\frac{X_{i}-\mathrm{mean}}{\mathrm{standard deviation}}\right)}^{4}-3$$

2 $${\mathrm{Sk}}_{2}=\frac{3\left(\mathrm{mean}-\mathrm{median}\right)}{\mathrm{standard deviation}}$$

Phase purity and crystallinity of the synthesized Mg:RuBisCO complexes were evaluated using powder X-ray diffraction (P-XRD) on a PANalytical X’Pert Pro X-ray diffractometer (Malvern Panalytical, United Kingdom) with Cu Kα radiation operating at 40 kV and 40 mA. Each P-XRD scan was set at a range from 4.99° to 69.96° with the step size of 0.0083556° and time per step of 40.005 s, respectively. Crystal structure and peak index analyses from P-XRD were performed by using HighScore Plus v3.0 P-XRD collection; sample evaluation was repeated at least 3 times for each ratio and its corresponding synthesis time, respectively. All of the available peaks were automatically indexed, including their intensity, location, and profile width and shape, respectively, against the background curve. Identified peaks were searched and matched based on the powder diffraction file (PDF) patterns maintained by the International Center for Diffraction Data (ICDD), yielding a list of possible candidates with the matching score based on the higher score, and the more likely the structure of the sample based on the identifiable peaks and (*hkl*) automatically calculated. The unit cell parameters were based on the only structure that was capable of being identified at the highest score defined by the software.

XRD, FTIR, and particle size analysis graphs were plotted by using OriginPro.

### 2.4 N_2_ Isotherm and CO_2_ Adsorption Studies

Synthesized Mg:RuBisCO complexes, specifically 50:1 and 100:1, were evaluated for their capability to absorb N_2_ and CO_2_ gases following a similar established protocol.^120^ Briefly, prior to N_2_ and CO_2_ gas adsorption, ∼500 mg of the respective sample was transferred to a round-bottom gas dispersion tube and degassed with N_2_ at 35 °C for at least 24 h to remove any possible moisture and contaminants. The weights, both prior to degassing and after degassing, were recorded into the Micromeritics proprietary software for data collection and analysis, respectively. For physisorption, the 50:1 and 100:1 Mg:RuBisCO complexes synthesized at 12 h were tested for N_2_ and CO_2_ gas adsorption on a Micromeritics ASAP 2060 apparatus (Micromeritics, Georgia, USA). For this, the round-bottomed gas dispersion tube containing the degassed sample was inserted into the sampling port of the ASAP 2060 instrument. To ensure the same temperature inside and outside of the sample tube, a chiller dewar of liquid solution was set on the outside of the tube. For N_2_ physisorption, a temperature-controlled dewar containing ethylene glycol was used. Vacuum was pulled from the tube, and the tube, subsequently dosed with the targeted gas incrementally pressurized for adsorption data analysis.

The 100:1, 12 h Mg:RuBisCO and CO_2_ static chemisorption was performed at 25, 50, and 75 °C with a Micromeritics ASAP 3Flex (Micromeritics, Georgia, USA). Following sample preparation similar to that for the physisorption testing, ∼200 mg of sample was transferred into a specially designed glass tube. The degassed sample inside the tube was sandwiched between 2 cotton balls. The weight of the sample, tube, and tube with sample were recorded manually and then input into the Micromeritics Flex proprietary software. The glass tube was inserted into the sample port of the instrument. A thermocouple and a furnace were used to heat the tube to the desired temperature for testing. Static chemisorption tested for the adsorption of the sample involved a predetermined pressure increment. Once the final pressure was achieved, the sampling tube was depressurized back to vacuum.

In order to evaluate the chemical adsorption capability of CO_2_ with Mg:RuBisCO, in situ KBr FTIR was also employed by using a transmission cell.^121^ The cell was placed between the IR source and the DTGS KBr detector in a Nicolet iS50 FTIR instrument. The material of interest, in the form of a paper-thin pellet, was secured inside the cell. Specifically, the Mg:RuBisCO-KBr pellet was obtained by mixing ∼100 mg of 100:1 12 h Mg:RuBisCO with ∼100 mg of KBr, followed by hydraulic pressing into a paper-thin pellet. A background with only a KBr paper-thin pellet was established, followed by analysis of the Mg:RuBisCO–KBr pellet. The FTIR spectra were taken after the chamber was purged with 5 mL/min of He for 2 h before operation to ensure an inert environment and, then, taken again with 10 mL/min of CO_2_ for 1, 5, 10, 15, and 20 min, respectively.

The N_2_ and CO_2_ physisorptions, CO_2_ static chemisorption, and in situ KBr FTIR testing were repeated 3 times. Post-data analysis including the Brunauer–Emmett–Teller (BET) surface area, Langmuir surface area, and pore volume was performed on the respective Micromeritics proprietary software and plotted using OriginPro. Briefly,

3 $$y=\frac{1}{Q\left(P_{0}/P-1\right)}$$

4 $$\frac{P/P_{0}}{Q\left(1-P/P_{0}\right)}=\frac{1}{Q_{m,\mathrm{BET}}C}+\frac{C-1}{Q_{m,\mathrm{BET}}C}\left(P/P_{0}\right)$$

5 $$S_{\mathrm{BET}}\left(\frac{m^{2}}{g}\right)=\sigma_{N_{2}}N_{A}\frac{Q_{m}}{V_{N_{2}}}=4.36\left(Q_{m,\mathrm{BET}}/\left(\mathrm{mL}\left(\mathrm{STP}\right)\right)\right)$$

Equation 3 shows the BET plot transformation from the adsorption isotherm data, with *P*/*P*_0_ presented as the relative pressure between the equilibrium pressure (*P*) and the saturation pressure (*P*_0_) and *Q* as the specific amount of gas adsorbed by the material. In some published literature, *Q* was typically reported interchangeably as *V* ^122^ or *n*,^123,124^ and *P*/*P*_0_ as *p*/*p* ^0^.^123^ When evaluating BET transformation in the linear form (*y* = *mx* + *b*), eq 4 defined *Q*_m,BET_) as the specific monolayer capacity/volume calculated using the BET method and *C* was related to the energy of monolayer adsorption. Once *Q*_m,BET_ was determined, the BET surface area (*S*_BET_) can be calculated using eq 5,^120,125,126^ where σ_N_2__ is the cross-section of the N_2_ equation (0.162 nm^2^ ^126^), *N*_A_ is the Avogadro number, and *V*_N_2__ is the molar gas volume of nitrogen (22400 cm^3^ (STP)), and it was noted that the unit of *Q*_m,BET_ here must be in mL (STP) or cm^3^ (STP).

Nonlocal density functional theory (NL-DFT) model of adsorption was used to characterize the pore size distribution with respect to the cumulative and differential pore volume, via the use of a N_2_ 77 K on carbon slit pores model provided by Micromeritics proprietary software. Briefly, the NL-DFT method utilized the classical fluid density functional theory to construct the adsorption isotherms in ideal pore geometries.^127,128^ NL-DFT model took account of the complex interactions^129–131^ (i.e., pore geometry^128^ and surface heterogeneity^132^) between the adsorbed gas molecules and pore walls, resulting as a better pore size characterization method with better precision for the micropores’ and smaller pores’ distribution of the order of 10 nm or lesser,^127,128,133,134^ as compared to the Barrett–Joyner–Halenda (BJH) model^133^ which yielded underestimations of micropores and small mesopores and therefore was not a suitable option when evaluating such region.^135,136^ The authors considered that the NL-DFT model is currently being recommended as a standard procedure by the International Organization for Standardization (ISO 15901-2:2022).^128,137^

## 3 Results

Room-temperature synthesis of metal–enzyme complexes was successfully achieved by incubating different ratios of purified enzymes and Mg-based salt under aqueous conditions. To verify the complex formation and characterize its physical and chemical properties, a range of analytical techniques was employed.

### 3.1 Microstructural and Compositional Evaluation of Metal–Enzyme Complexes

Initial screening of the synthesis conditions and analysis of the surface morphology were performed by using scanning electron microscopy (SEM). Qualitative results showed that different times of synthesis (i.e., 1, 3, 6, 12, and 24 h) and mass ratios of Mg:RuBisCO used for such synthesis (i.e., 5:1, 50:1, and 100:1), respectively, influenced resulting structure formations. For the 5:1 ratio, agglomeration of both irregular, shredded, or segmented morphologies and rhomboidal and cylindrical-shaped complexes with the degree of such shapes varying function of the synthesis time were observed. The generally disrupted, poorly organized structures (Figure 1a–e) were most likely indicati suboptimal aggregation of metal–enzyme complexes under these synthesis conditions. For the 50:1 ratio, more regular and uniform morphologies were observed, indicating a more effective organization of the metal–enzyme complexes. Briefly, the 1 and 3 h synthesis revealed mostly rhomboidal shapes of agglomerated complexes and a mixture of smaller, irregular shapes on or surrounding such structures (Figure 1f,g). At 6 and 12 h, the rhombic structures were shown to be larger in size (Figure 1h,i), with the addition of a lotus-like shape formation also observed (Figure 1i,j). For the 100:1 ratio, at 1 h (Figure 1k–o) time of synthesis, complexes were a combination of rhomboidal and cylindrical shapes, while what resembles a lotus-like form composed of rhomboidal structures also appeared after 3 h of reaction time. This morphology was preserved at both 12 and 24 h reaction times (Figure 1k). Control experiments with MgCl_2_ Supporting Information (SI) Figure S1 showed no structural densities.

**Figure 1:**
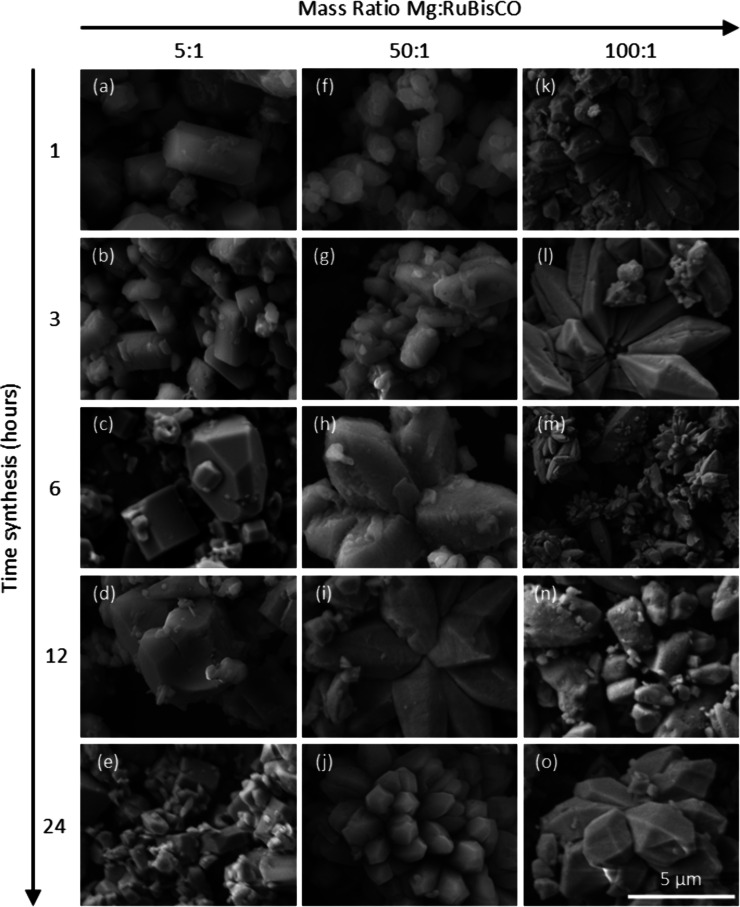
SEM images of complexes formed using different mass ratios (left–right) and time of reaction, respectively. Morphologies of complexes formed using the 5:1 ratio of Mg:RuBisCO are shown in panels a–e, morphologies of complexes formed using the 50:1 ratio of Mg:RuBisCO are shown in panels f–j, while morphologies of complexes formed using the 100:1 ratio of Mg:RuBisCO are shown in panels k–o, respectively. All images were captured at 12000× magnification. A 5 μm scale bar is shown at the bottom right corner of panel o.

To confirm metal incorporation and evaluate the elemental distribution in the complexes, energy-dispersive X-ray spectroscopy (EDX) was performed. Table S1 shows the elemental composition of the hybrids with average and standard deviation for each synthesis condition (i.e., 1, 3, 6, 12, and 24 h, respectively) with respect to the mass ratio (i.e., 5:1, 50:1, and 100:1) being used, while Figure S2 shows the graph analysis associated with these conditions. Elemental analysis revealed clear signals corresponding to Mg^2+^, thus supporting successful coordination with the enzyme and its incorporation into the complexes. Oxygen, carbon, and phosphorus were also observed as associated with the enzyme composition; no impurities were present.

### 3.2 Chemical and Structural Evaluation of Metal–Enzyme Complexes

To further confirm metal incorporation, the chemical characteristics of the complexes were investigated through Fourier transform infrared spectroscopy (FTIR) analysis (Figure 2). Briefly, by comparison, analysis showed that 24 h synthesis time led to a higher concentration of functional groups for both 50:1 and 100:1 ratios of Mg:RuBisCO relative to either 1.5:1 (which generally showed a flat profile) or 5:1 (which generally showed peaks of small intensity) ratio, respectively. Specifically, in the complexes formed at a low ratio of metal to enzyme, the characteristic amide I (∼1650 cm^–1^) and amide II (∼1540 cm^–1^) bands of the enzyme remained largely unchanged compared to those of the native enzyme (Figure S3), suggesting that the reduced integration and binding of the metal ions did not significantly perturb the enzyme backbone.

**Figure 2:**
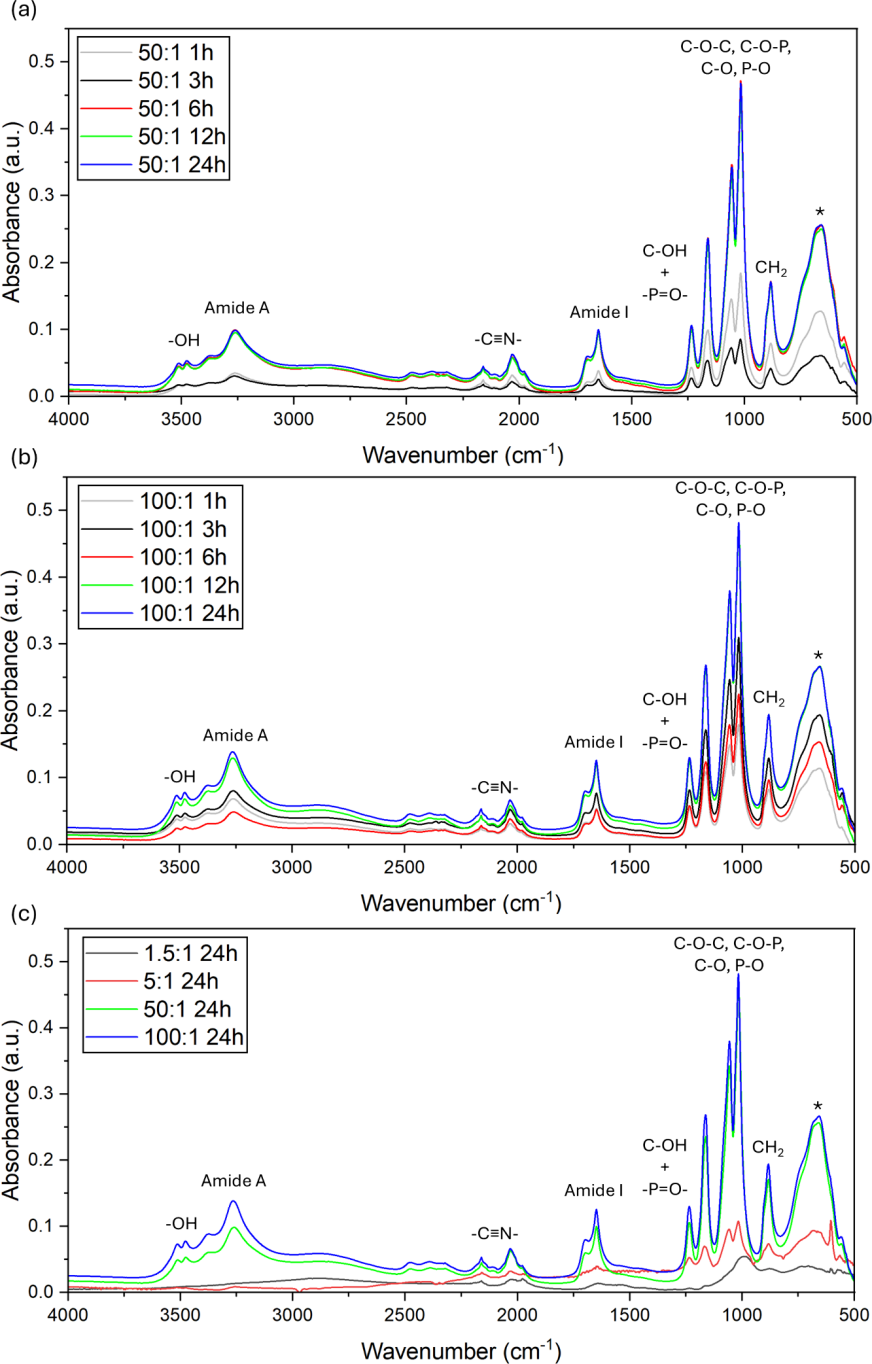
(a) ATR-FTIR of Mg:RuBisCO in 50:1 and (b) 100:1 ratios at different reaction times (i.e., 1, 3, 6, 12, and 24 h). (c) ATR-FTIR of Mg:RuBisCO of different synthesis ratios (1.5:1, 5:1, 50:1, and 100:1) at 24 h, respectively.

A broad peak of the absorption band was observed between 3000 and 3600 cm^–1^ for the complexes formed at 50:1 and 100:1 ratios, and it was attributed to the characteristic absorption of hydrogen bond vibration, with the hydroxyl (−OH) group^138,139^ being the most significant contributor at around 3518 cm^–1^ (specific peak location and functional groups are shown in Table 1 for both 50:1 and 100:1 ratios respectively). Other vibrations incurred from 3000 to 3700 cm^–1^ most likely associated with the primary amide groups^141–144^ due to the two −NH– stretching vibrations around 3366–3710 cm^–1^. The cyanide (−C≡N−) functional group^138,145^ was observed between 2162 and 2037 cm^–1^, while the amide I band was observed between 1700 to 1649 cm^–1^, as caused by the vibrations from C=O, C–N stretch, and/or N–H bending of the amino acids bonds.^141–144,146,147^ Characteristic peaks for phosphoester bond were present as sharp peaks at 1234 cm^–1^ for C–OH with P=O^148–150^ and at 1163, 1057, and 1016 cm^–1^ for aliphatic and aromatic C–O–C, C–O–P, C–O, and P–O,^150,151^ respectively. The peak at 883 cm^–1^ was attributed to the rocking vibration of CH_2_.^148,152^ Lastly, the broad band from 850 to 550 cm^–1^ was presumably due to the stretching vibrations of Mg–O–Mg as previously reported.^153–156^

**Table 1:** Peak’s Location and Functional Groups Identified from the FTIR Spectra for 50:1 and 100:1 Mg:RuBisCO Complexes

peaks range (cm^–1^)	functional group identifier	ref
3600 to 3000	hydroxyl (−OH)	^138^,^139^
3710 to 3370	primary amide (amide A)	^140–144^
2162 to 2037	cyanide (−C≡N−)	^138^,^145^
1700 to 1649	amide I (peptide bond)	,^147^
1234	C–OH + P=O	^148–150^
1163 to 1016	C–O–C + C–O–P + C–O + P–O	^150^,^151^
883	CH_2_ rocking	^148^,^152^
850 to 600	Mg–O–Mg	^153–156^

Following such initial screening of multiple metal–enzyme ratios and times of synthesis, respectively, only those yielding well-defined morphologies (as supported by SEM imaging) and chemical composition confirming metal integration (as supported by FTIR analysis) were used for additional structural and functional characterization. Specifically, particle size distribution analysis was evaluated as the dispersion quality of the samples at different ratios and time points of synthesis, respectively. While SEM provided high-resolution qualitative imaging of surface features, particle size analysis offered quantitative insights into the overall size and polydispersity of the complex’s suspensions. Particle size analysis is shown in Figure 3 with supporting evaluation shown in Table 2 and Table 3 for the 50:1 and 100:1 ratios, respectively.

**Figure 3:**
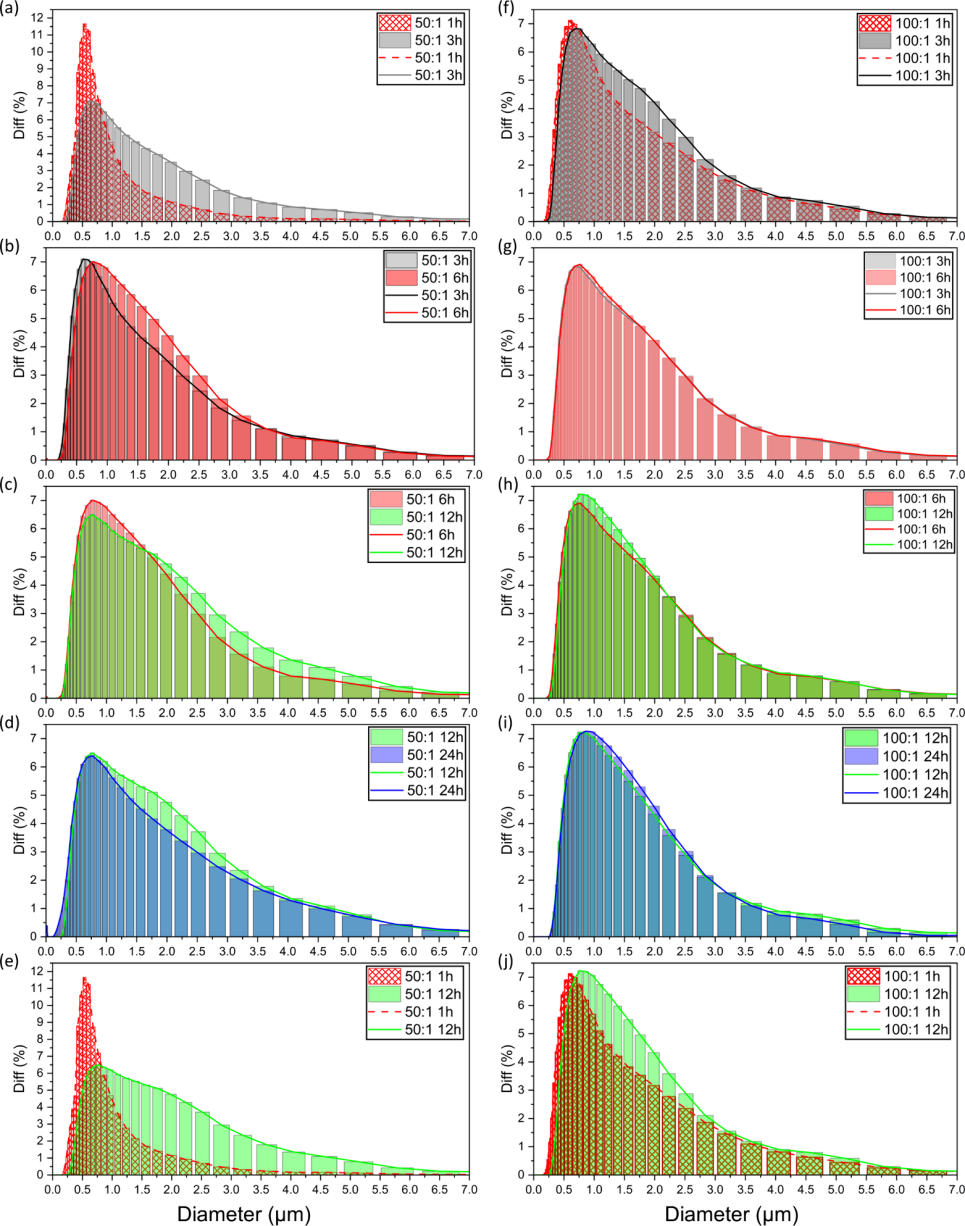
Laser diffraction particle size analysis (*n* = 15) for (left) 50:1 Mg:RuBisCO and (right) 100:1 Mg:RuBisCO; (top-bottom) comparison between each time of the synthesis intervals, namely, (a, f) 1 vs 3 h, (b, g) 3 vs 6 h, (c, h) 6 vs 12 h, (d, (i) 12 vs 24 h, and (e, j) 1 vs 12 h, respectively.

**Table 2:** Mean (μm), Median (μm), Mode (μm), Standard Deviation (μm), Kurtosis, and Pearson Skewness Coefficient for 50:1 Mg:RuBisCO Complexes at 1, 3, 6, 12, and 24 h (*n* = 15) of Synthesis

param	1 h	3 h	6 h	12 h	24 h
mean (μm)	0.62 ± 0.07	0.88 ± 0.12	0.99 ± 0.074	1.07 ± 0.043	1.00 ± 0.23
median (μm)	0.57 ± 0.055	0.80 ± 0.118	0.94 ± 0.07	1.01 ± 0.054	0.96 ± 0.21
mode (μm)	0.52 ± 0.046	0.62 ± 0.074	0.76 ± 0.092	0.70 ± 0.062	0.75 ± 0.16
std dev (μm)	0.23 ± 0.066	0.3 ± 0.029	0.28 ± 0.045	0.29 ± 0.016	0.31 ± 0.037
kurtosis	5.65	3.24	3.86	2.61	3.22
skewness coefficient	0.60	0.79	0.53	0.62	0.45

**Table 3:** Mean (μm), median (μm), mode (μm), Standard Deviation (μm), Kurtosis, and Pearson Skewness Coefficient for 100:1 Mg:RuBisCO Complexes at 1, 3, 6, 12, and 24 h (*n* = 15) of Synthesis

param	1 h	3 h	6 h	12 h	24 h
mean (μm)	0.83 ± 0.17	0.96 ± 0.088	0.97 ± 0.077	1.01 ± 0.055	1.01 ± 0.044
median (μm)	0.76 ± 0.17	0.90 ± 0.097	0.91 ± 0.085	0.95 ± 0.05	0.97 ± 0.045
mode (μm)	0.58 ± 0.083	0.68 ± 0.096	0.71 ± 0.068	0.77 ± 0.094	0.81 ± 0.085
std dev (μm)	0.30 ± 0.032	0.28 ± 0.006	0.29 ± 0.013	0.27 ± 0.034	0.26 ± 0.02
kurtosis	3.92	3.08	3.42	3.36	3.24
skewness coefficient	0.72	0.62	0.62	0.62	0.46

Both 50:1 and 100:1 Mg:RuBisCO ratios reflected a broad positive skew unimodal distribution, with longer times of synthesis shifting the distribution toward bigger size. Specifically, at 1 vs 3 h synthesis for the 50:1 Mg:RuBisCO ratio, there was a 43% significant increase in mean (from 0.62 ± 0.07 to 0.88 ± 0.12 μm), 41% in median (from 0.57 ± 0.055 to 0.8 ± 0.12 μm), 18% in mode (from 0.52 ± 0.046 to 0.62 ± 0.074 μm), and 31% in standard deviation (from 0.23 ± 0.066 to 0.3 ± 0.029 μm), respectively, suggesting a rapid growth and/or agglomeration of complexes in these conditions. Complementarily, the kurtosis value decreased from 5.7 to 3.2, suggesting that the peak was flattening out with the increase in the time of synthesis, where larger size complexes were forming in a more broad distribution at 3 h as compared to the sharp peak and narrow distribution at 1 h. The skewness coefficient also showed an increase from 0.6 to 0.79, indicating that complexes at 3 h were becoming larger, as shown by the positive skewness plot with a larger and longer tail (Figure 3a).

From 3 to 6 h synthesis time and for the 50:1 Mg:RuBisCO ratio (Figure 3b), an increase in the particle size and size distribution was again observed, however at a slower rate, with a mean size increase of 12% (from 0.88 ± 0.12 to 0.99 ± 0.074 μm), a median size increase of 17% (from 0.80 ± 0.12 to 0.94 ± 0.07 μm), and a mode increase of 22% (from 0.62 ± 0.074 to 0.76 ± 0.092 μm), respectively. Standard deviation decreased slightly (by 5.6%), suggesting that the spread of the complexes’ distribution was not changing significantly; the kurtosis value increased slightly from 3.2 to 3.9, implying that the complex sizes were concentrating around the mean and median values. The skewness coefficient decreased significantly from 0.79 to 0.53, suggesting that the influence of larger complexes at 6 h was becoming less pronounced relative to that of complexes at 3 h, also presumably indicating that the growth of larger structures slowed down, i.e., reaching a growth phase plateau.

From 6 to 12 h and for the 50:1 Mg:RuBisCO ratio (Figure 3c), the mean and median sizes for 12 h complexes showed a continued increase relative to the same values at the 6 h synthesis time, with a 8% increase (from 0.99 ± 0.074 to 1.1 ± 0.043 μm) and 7% (from 0.94 ± 0.07 to 1 ± 0.054 μm), respectively. However, the mode decreased by 7% (from 0.76 ± 0.092 to 0.7 ± 0.062 μm), presumably suggesting a shift in the population of complexes. The value of kurtosis also decreased from 3.86 to 2.61, indicating that the distribution of such complexes was flattened and spread out broadly. Contrarily, the skewness coefficient increased from 0.53 to 0.62, showing that the tail for the 12 h complexes’ distribution was becoming larger presumably due to the continued aggregation of such structures. Overall, between 6 and 12 h, there was a phase of continued growth with a potential shift in the primary solution population toward slightly smaller sizes, specifically at 0.7 ± 0.062 μm, and with a broader range of larger structures.

For the 50:1 Mg:RuBisCO ratio (Figure 3d), the mean and median sizes for 24 h complexes decreased by 6% (from 1.1 ± 0.043 to 1 ± 0.231) μm) and 5% (from 1 ± 0.054 to 0.96 ± 0.21 μm), respectively. The mode size of 24 h synthesized complexes increased by 7.1% (from 0.7 ± 0.062 to 0.75 ± 0.16 μm). As shown in Figure 3, the larger and broader tail observed on the right side of the distribution at 12 h started to flatten out as the tail on the left side of the distribution started to increase; hence, the standard deviation increased from 0.29 to 0.31. The kurtosis value increased from 2.6 to 3.2, and the skewness coefficient decreased significantly from 0.62 to 0.45. When comparing 1 to 12 h synthesis for 50:1 Mg:RuBisCO, the process showed significant growth, whereas the entire size distribution of the structures shifted to a larger diameter to become substantially broader, with a bigger and longer tail appearing on the right side of the distribution as the synthesis time increased (Figure 3e).

For 100:1 Mg:RuBisCO at 1 to 3 h synthesis time (Figure 3f), there was an increase of 16% for the mean (from 0.83 ± 0.17 to 0.96 ± 0.088 μm), 19% for the median (from 0.76 ± 0.17 to 0.9 ± 0.097 μm), and 16% (from 0.58 ± 0.083 to 0.68 ± 0.096 μm) for the mode, respectively. This pattern was similar to the 50:1 ratio, indicating that the complex size was increasing as the time of synthesis increased; however, the growth seemed to be less dramatic relative to the 50:1 Mg:RuBisCO ratio at 1 and 3 h, respectively. Standard deviation of samples decreased slightly from 0.3 ± 0.032 to 0.28 ± 0.006 μm, with the skewness coefficient also decreasing from 0.72 to 0.62, suggesting that the distribution at 3 h was becoming more concentrated within a range of sizes of larger dimensions. The kurtosis value decreased from 3.92 to 3.1, suggesting that the concentration of complexes for the 3 h time of synthesis was spreading out as the peak was smaller and broader at the center of the distribution.

From 3 to 6 h at 100:1 Mg:RuBisCO ratio (Figure 3g), the mean size increased by 1% (from 0.96 ± 0.088 to 0.97 ± 0.077 μm), while the median decreased by 1% (from 0.90 ± 0.097 to 0.71 ± 0.068 μm). Complementarily, the mode showed a 35% increase from 0.68 ± 0.096 to 0.91 ± 0.085 μm, while the standard deviation showed little to no change in value, i.e., from 0.28 ± 0.006 to 0.29 ± 0.013 μm. The kurtosis value presented a slight increase from 3.1 to 3.4, with the skewness coefficient also presenting a slight increase, suggesting that the 6 h time of synthesis was a continuation of growth, yet at a significantly slower rate relative to that recorded for the 1 or 3 h synthesis, respectively.

Analysis for the 6 compared to 12 h (Figure 3h) synthesis times also reflected an increase in mean of 4% (from 0.97 ± 0.077 to 1.0 ± 0.055 μm), in median of 5% (from 0.91 ± 0.085 to 0.95 ± 0.05 μm), and in mode of 8% (from 0.71 ± 0.068 to 0.77 ± 0.094 μm). Meanwhile, the standard deviation of samples at 12 h decreased by 5% (from 0.29 ± 0.013 to 0.27 ± 0.034 μm), indicating that the distribution of sizes became narrower. The skewness coefficient presented no change, while the value of kurtosis showed very minimal to no significant decrease. Around 12 h, it was also shown that the growth of complexes seemed to have continued, with the mean, median, and mode all recording an increase, however with the distribution at the two ends of the tails not changing drastically.

The 100:1 Mg:RuBisCO ratio at 24 h relative to the 12 h synthesis time (Figure 3i) showed that the mean size increased slightly but not significantly with specific values ranging from 1.01 ± 0.055 to 1.01 ± 0.044 μm. Median size also appeared to increase slightly by 2% from 0.95 ± 0.05 to 0.97 ± 0.045 μm, while the mode increased by 6% (from 0.77 ± 0.094 to 0.81 ± 0.085 μm). Standard deviation decreased by 4% (from 0.27 ± 0.034 at 12 h to 0.26 ± 0.020 μm at 24 h), hence showing a narrower and more uniform distribution of such sizes. The kurtosis value also decreased slightly from 3.4 to 3.24, possibly indicating that the concentration of complexes was around the mode as the distribution narrowed. The skewness coefficient decreased significantly from 0.62 to 0.46, suggesting that more uniform distributions of sizes were achieved. The synthesis between 12 and 24 h also showed a stabilization of the sizes, where larger complexes were deagglomerated; hence, the distribution shifted the mode and median toward larger size with its tail on the right side of the distribution decreased. Overall, for the 100:1 Mg:RuBisCO complexes, there was an obvious shift of the entire distribution to larger dimensions from 1 to 12 h (Figure 3j), as recorded in both Tables 2 and 3 respectively.

X-ray diffraction (XRD) was conducted to complement SEM and size distribution analysis and to assess the crystallinity of the metal–enzyme complexes; results are shown in Figure 4 for powder diffractograms of 50:1 and 100:1 Mg:RuBisCO ratios, at different time points, all relative to the controls salt and enzyme, respectively. Briefly, diffractograms confirmed the formation of Mg:RuBisCO complexes with clear structural integrity relative to controls, with the XRD peaks revealing the formation of a polycrystalline structure with several distinct peaks. Peaks are very similar (in terms of their overall aspect ratio and distribution, respectively) between the two ratios used for synthesis, namely, 50:1 and 100:1. Furthermore, no unusual large broadening was observed, thus indicating that the synthesized Mg:RuBisCO complexes were of high purity. Closer evaluation, however, identified systematic changes in the position of some of the diffraction peaks (2θ) function of the synthesis time and ratio. Specifically, across all time points, the 50:1 ratio presented a noticeable peak shift toward the higher 2θ angles as the time of a complex’s synthesis increased (Figure 5), while for the 100:1 ratio such changes were of smaller shift toward the lower 2θ angles, again as time of synthesis increased (Figure 6).

**Figure 4:**
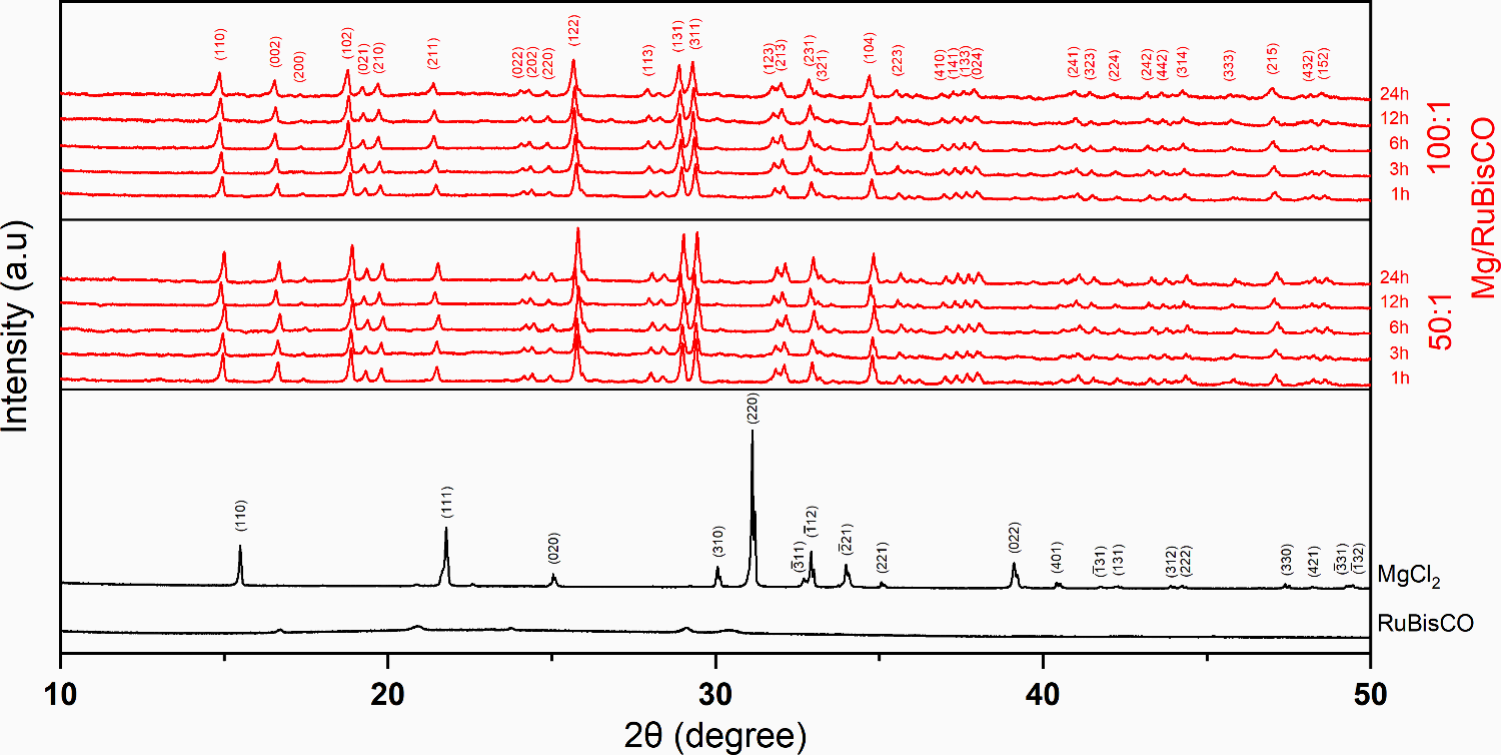
XRD of (black) RuBisCO, (black) MgCl_2_, and (red) all time points of 50:1 and 100:1 Mg:RuBisCO complexes, respectively.

**Figure 5:**
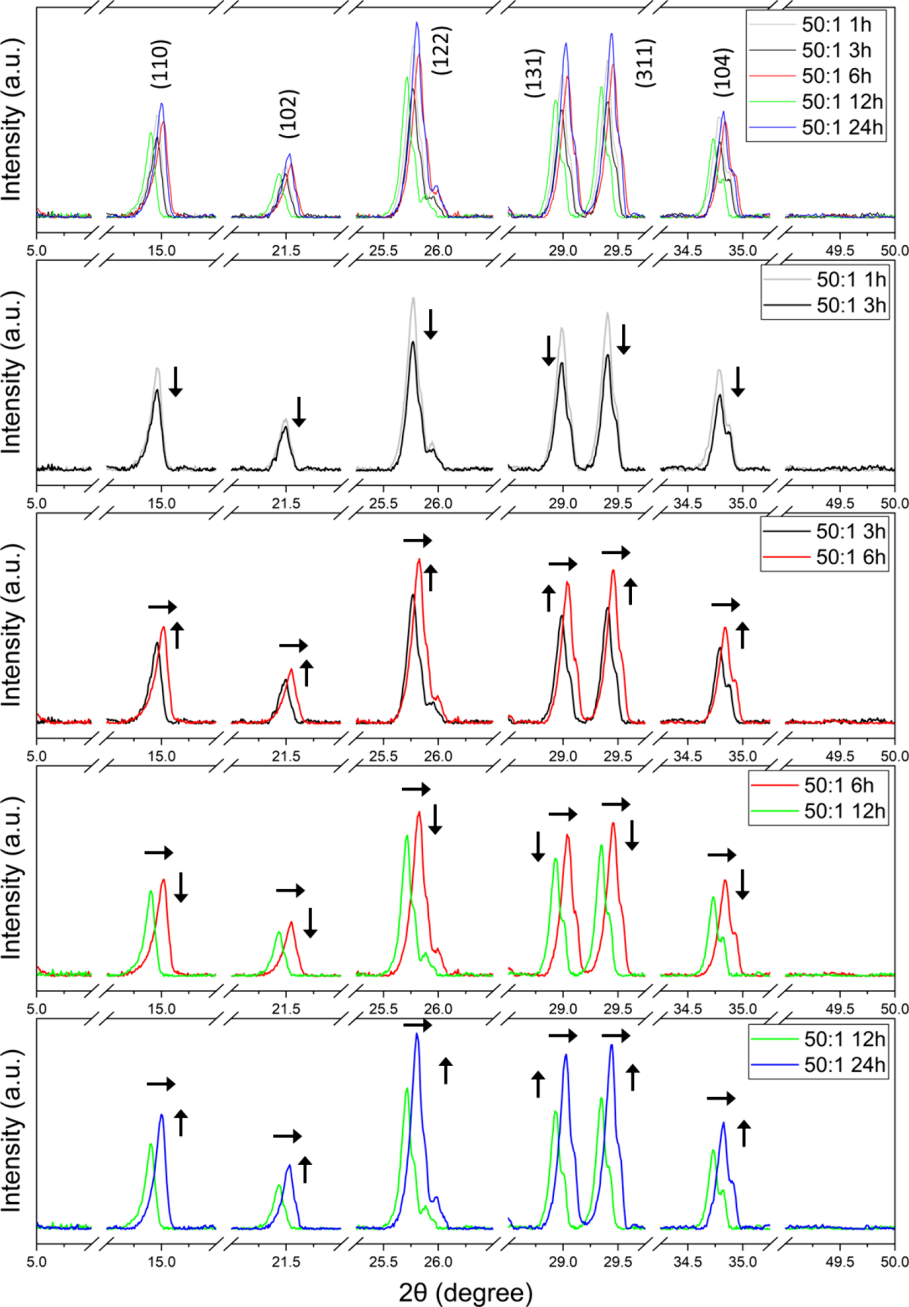
Comparisons of all time points of synthesis for the 50:1 ratio of the Mg:RuBisCO complex. Arrows indicate the shifts in peaks, either in intensity or localization.

**Figure 6:**
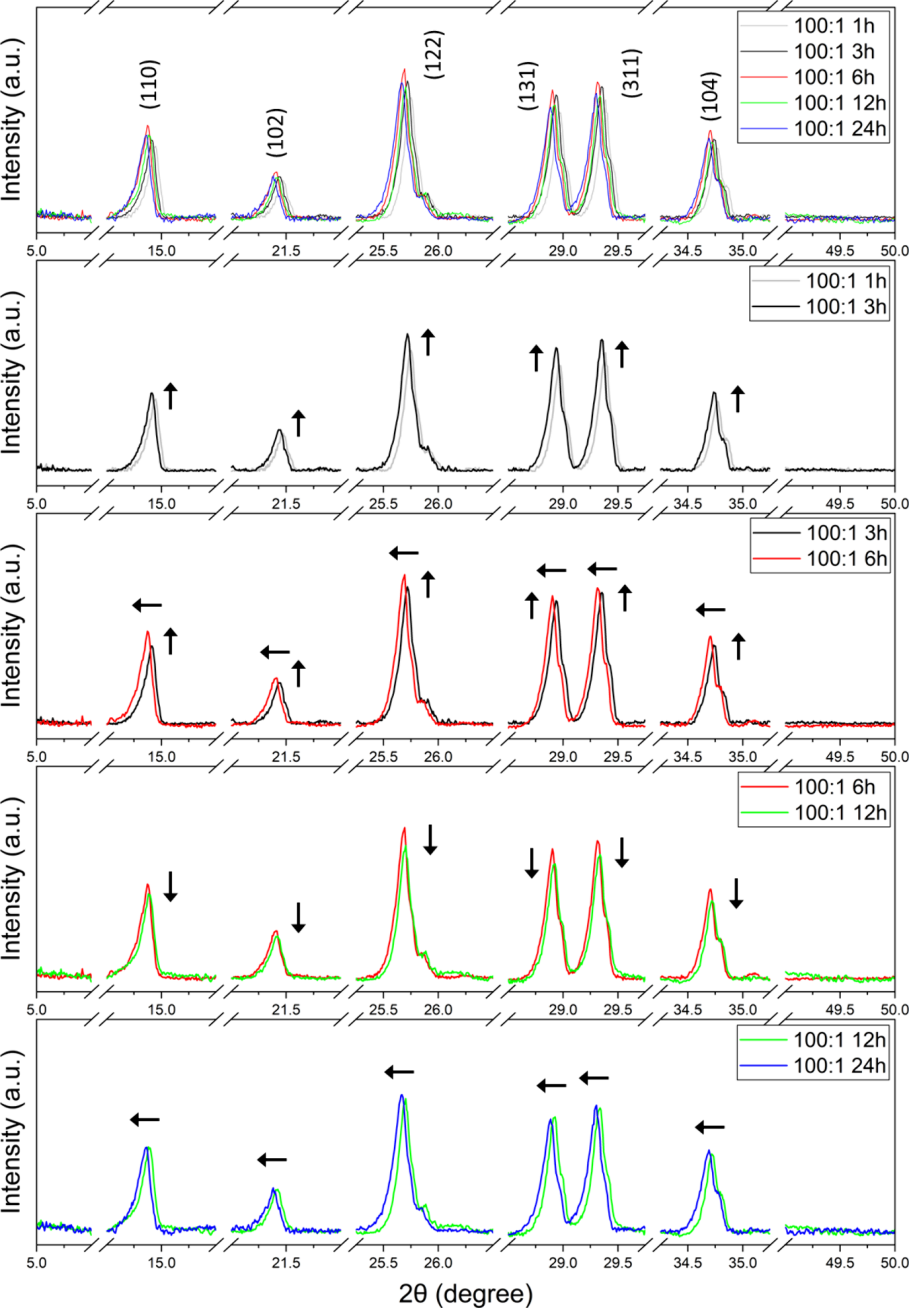
Comparisons of all time points of synthesis for the 100:1 ratio of the Mg:RuBisCO complex. Arrows indicate the shifts in peaks, either in intensity or localization.

The diffraction peaks were also indexed based on standard reference data, and the corresponding 2θ positions, interplanar spacings (*d*, in ångströms), full width at half-maxima (fwhm) relative intensities, and (*hkl*) planes were identified using the X’Pert HighScore Plus software, with results shown in Table 4 for the complexes and in Table S2 for the control, respectively. Analysis indicated the presence of ordered domains presumably arising from the coordination between the enzyme and metal ions, with the enzyme itself most likely serving as a scaffold. The most intense reflections, i.e., where the diffraction peak had the highest relative intensity in the XRD pattern, were associated with the (110), (102), and (122) planes, all with relative intensities higher than 90%. The (102) plane appeared to dominate the orientation of crystallites, as evidenced by the intensity of its plane. The overall high intensity observed for the (110), (102), and (122) planes indicated the highly preferred orientation and partial crystallinity of the complex with some alignment of these crystallographic planes likely to be parallel to the sample surface. Relative intensities higher than 60% were observed for (002), (131), and (311) planes, while many of the other peaks displayed significantly lower (mostly in the 10–40% range) relative intensities. Contrary, analysis of the salt used as a control showed the most intense reflections for the dominant (111) plane (Table S2).

**Table 4:** Diffraction Data of Mg:RuBisCO, Including Peak Index Based on the 2θ Location, *d*-Spacing (Å), Relative Intensity, and *hkl* Indices of Each Peak

2θ (deg)	*d*-spacing (Å)	relative intensity (%)	indices (*hkl*)
14.94	5.92	97.47	(110)
16.62	5.33	65.52	(002)
17.41	5.09	9.71	(200)
18.85	4.70	100	(102)
19.30	4.60	30.81	(021)
19.78	4.49	40.56	(210)
21.47	4.14	40.94	(211)
24.14	3.68	13.81	(022)
24.39	3.65	15.62	(202)
24.94	3.57	15.33	(220)
25.75	3.46	94.96	(122)
28.01	3.18	18.28	(113)
28.97	3.08	67.18	(131)
29.39	3.04	79.70	(311)
31.81	2.81	21.35	(123)
32.08	2.79	32.80	(213)
32.93	2.72	42.01	(231)
33.16	2.70	9.18	(321)
34.77	2.58	45.12	(104)
35.60	2.52	13.38	(223)
36.99	2.43	8.68	(410)
37.34	2.41	13.69	(141)
37.66	2.39	17.00	(133)
37.96	2.37	14.31	(024)
40.57	2.22	1.34	(241)
41.07	2.20	6.06	(323)
42.24	2.14	7.66	(224)
43.25	2.09	11.18	(242)
43.70	2.09	8.85	(422)
44.33	2.04	16.05	(314)
45.82	1.98	3.18	(333)
47.08	1.93	19.36	(215)
48.24	1.89	10.47	(432)
48.60	1.87	10.16	(152)

### 3.3 Gas Adsorption Studies Performed on Metal–Enzyme Complexes

Nitrogen (N_2_) adsorption experiments were conducted to determine the adsorption capacity, pore size, and pore volume distribution, respectively, of the synthesized complexes. Figure 7a presents the N_2_ adsorption for both 50:1 and 100:1 ratios at 12 h of synthesis; data showed the relationship between the relative pressure (*P*/*P*_0_) and the volume of N_2_ adsorbed at 77 K, with analysis revealing that complexes exhibited a Type II isotherm with a Type H3 hysteresis loop based on the International Union of Pure and Applied Chemistry (IUPAC)’s isotherm classification.^123^ Figure S4 shows the IUPAC classification for the (a) physisorption isotherm,^123^ (b) hysteresis loop,^123^ and (c) pores sizes based on pore width with a schematic ion diffusion pattern in a porous structure,^157^ respectively. Closer evaluation at pressures under 300 mmHg (*P*/*P*_0_ < 0.40) showed the “knee” or point B of the isotherm at ∼10–20 mmHg (*P*/*P*_0_ = ∼0.20–0.25),^123^ most likely indicating N_2_ monolayer coverage of the complexes upon gas adsorption. Further, the sharp increase in adsorption at low relative pressures (*P*/*P*_0_ < 0.2) was presumably due to a well-developed microporous structure, whereas the gradual uptake observed at higher relative pressures suggested that the formed complex possessed the larger end of a microporous structure with a combination of the smaller ends of a mesoporous domains, with similar observations in the N_2_ adsorption isotherm at lower pressures presented in other works.^158–162^

**Figure 7:**
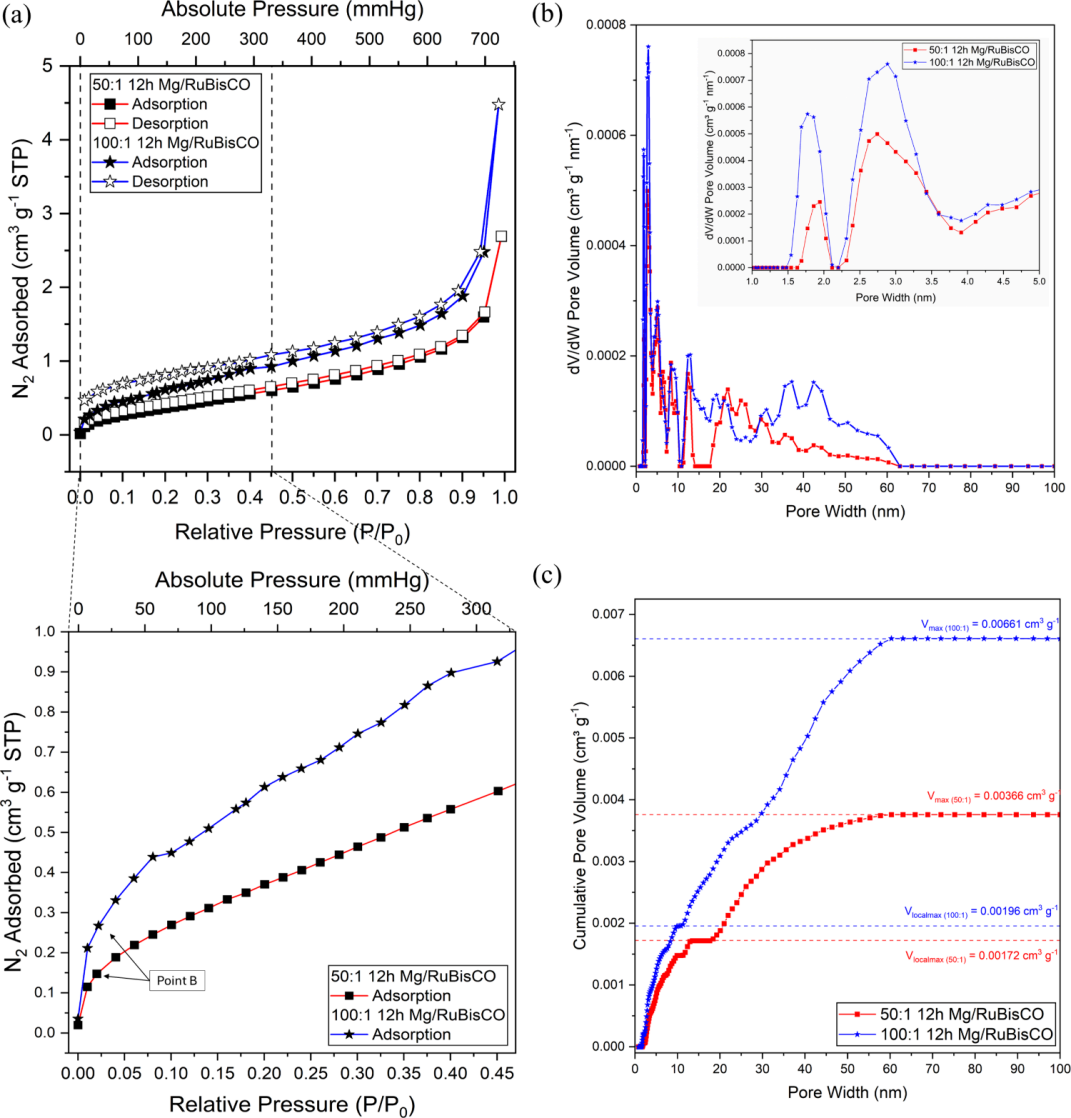
(a) N_2_ adsorption–desorption isotherm with (exploded) zoom-in view within the *P*/*P*_0_ range from 0 to 0.45, (b) NL-DFT differential pore volume with (inset) zoomed-in view of pore width from 1 to 5 nm, and (c) NL-DFT cumulative pore volume for a respective (red, square) 50:1 12 h Mg:RuBisCO and a respective (blue, star) 100:1 12 h synthesis time of Mg:RuBisCO complexes.

Indeed, the NL-DFT model of adsorption used to characterize the pore size distribution with respect to the differential (Figure 7b) and the cumulative pore volume (Figure 7c) respectively revealed that complexes have a heterogeneous pore size distribution consisting of a combination of micro- and mesopores. Specifically, for the 50:1 ratio and 12 h of synthesis, the complexes presented one broad peak in the micropore region centered at 1.9 ± 0.0024 nm and several broad peaks in the mesopore regions centering at 2.7 ± 0.018, 5.1 ± 0.0011, 6.7 ± 0.0004, 8.3 ± 0.0011, 13 ± 0.211, and 22 ± 0.179 nm, respectively. For the 100:1 ratio and 12 h of synthesis, the complexes also presented one broad peak in the micropore region centered at 1.8 ± 0.0008 nm and several broad peaks in the mesopore regions centered at 2.9 ± 0.064, 5.1 ± 0.036, 8.6 ± 0.0021, 12.9 ± 0.27, 20 ± 1.6, 37 ± 1.7, and 42 ± 3.1 nm, respectively. Peak intensity for the 100:1 ratio, 12 h Mg:RuBisCO within the width < 5 nm region was substantially higher than that for the 50:1, 12 h Mg:RuBisCO complexes, with an increase of 66% for pores within 1.8 nm and 55% for pores within 2 nm, signifying a more developed porous structure with much greater abundance of micropores for the higher Mg^2+^ concentration sample being synthesized. Additionally, at higher pore size, several new peaks were observed emerging in the region of 35–45 nm for the 100:1 12 h Mg:RuBisCO when compared to the same time of synthesis but the 50:1 ratio (Figure 7b). The pore size distribution in the mesopore region (width = 2–50 nm) calculated by NL-DFT from the N_2_ adsorption isotherm for a respective (red, square) 50:1 12 h Mg:RuBisCO and a respective (blue, star) 100:1 12 h Mg:RuBisCO sample is also shown in Figure S5.

The cumulative pore volume (i.e., the total volume of pores across different micropores and mesopores sizes) for the 50:1 ratio, 12 h synthesis of Mg:RuBisCO complexes was 0.0037 ± 0.00002 cm^3^ g^–1^ (*n* = 3) while for the 100:1 ratio it was 0.0066 ± 0.0003 cm^3^ g^–1^ (*n* = 3). Interestingly, a local maximum, where the cumulative pore volume graph plateaued briefly, was observed within the 10 – 20 nm region. These local maxima did not present a drastic change, with the recorded values being 0.0017 ± 0.00041 and 0.002 ± 0.0001 cm^3^ g^–1^ for 50:1 and 100:1, 12 h synthesis time Mg:RuBisCO, respectively. The drastic increase in volume happened when the pore sizes were >30 nm, which could have been a direct result of the accumulation of the emerging bigger pore sizes presented in the higher Mg^2+^ concentration samples as described above.

To accurately determine the specific surface area of the metal–enzyme composites (*S*_BET_), the Rouquerol–BET method^122,123,126,163–167^ was used; 0.05 < *P*/*P*_0_ < 0.35 range (where (*P*) is the equilibrium pressure and (*P*_0_) is the saturation pressure) was evaluated to avoid subjectivity in *Q*_m_ analysis, i.e., the monolayer adsorption capacity/volume (Table S3). The regression analysis of the Rouquerol criteria, which suggest having a linear range spanning at least 10 data points and *R* ^2^ ≥ 0.995,^164,166^ was enforced. Results for 50:1 and 100:1 12 h synthesized Mg:RuBisCO composites with the Rouquerol–BET plot are shown in Figure 8 with respective surface areas and porosity characteristics in Table 5.

**Figure 8:**
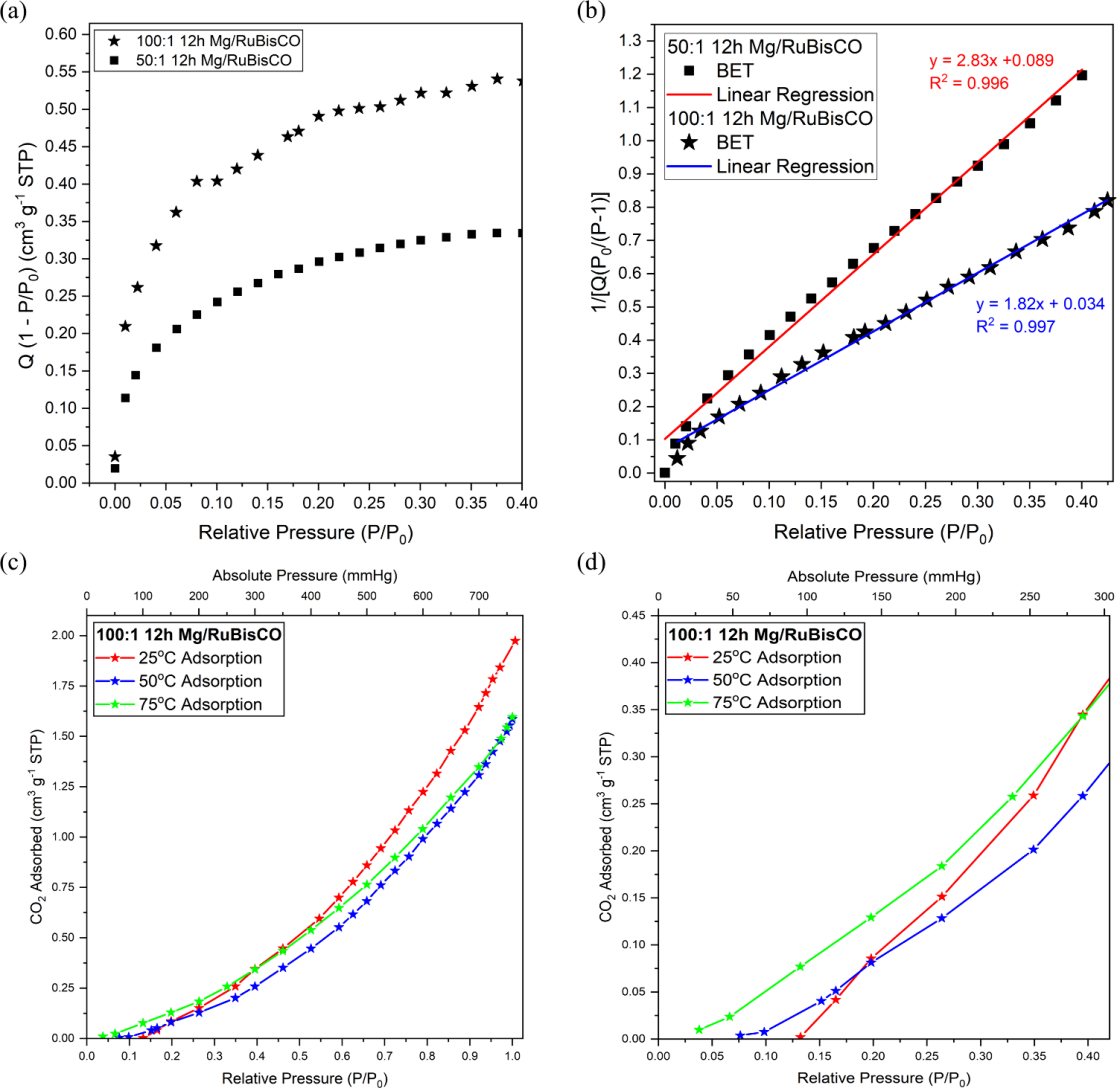
(a) Rouquerol–BET plot and (b) BET range corresponding to the satisfied Rouquerol criteria shown as linear regression for the representative (square) 50:1 and 100:1, 12 h synthesized Mg:RuBisCO complexes. (c) Full-view and (d) zoomed-in view for *P*/*P*_0_ from 0 to 0.4 for the CO_2_ adsorption of a representative 100:1 12 h Mg:RuBisCO complex at (black) 25 °C, (red) 50 °C, and (blue) 75 °C, respectively.

**Table 5:** *S*_BET_ (m^2^/g), *C*, *Q*_m,BET_ (cm^3^/g (STP)), Slope (g/cm^3^ (STP)), *y*-Intercept (g/cm^3^ (STP)), and *R* ^2^ Calculations Based on the Rouquerol–BET Model for 50:1 and 100:1 12 h Synthesized Mg:RuBisCO Complexes (*n* = 3)

ratio Mg:RuBisCO, time (h)	*S*_BET_ (m^2^/g)	*C*	*Q*_m,BET_ (cm^3^/g (STP))	slope (g/cm^3^(STP))	*y*-intercept (g/cm^3^(STP))	*R* ^2^
50:1, 12	1.5 ± 0.031	33 ± 0.014	0.34 ± 0.0062	2.83 ± 0.059	0.089 ± 0.014	0.995 ± 0.00031
100:1, 12	2.3 ± 0.0255	54 ± 0.02	0.54 ± 0.0025	1.82 ± 0.02	0.035 ± 0.004	0.997 ± 0.00014

Upon satisfying the Rouquerol criteria via Rouquerol–BET plot (Figure 8a) the linearized BET analysis data showed that the *R* ^2^ value was ≥0.995; specifically, *R* ^2^ = 0.995 for 50:1 12 h Mg:RuBisCO complexes and *R* ^2^ = 0.997 for 100:1 12 h Mg:RuBisCO complexes. Subsequently, the calculation of the subsequent *Q*_m_, *C*, and *S*_BET_ values based on the slope and *y*-intercept from the linearized BET plot, within the satisfied *P*/*P*_0_ range, showed that 100:1 12 h Mg:RuBisCO complexes had an increase in BET surface area of ∼57% from 1.5 to 2.3 m^2^/g and an increase in the monolayer capacity *Q*_m_ also by ∼57% from 0.34 to 0.54 cm^3^/g (STP), when compared to the 50:1 12 h Mg:RuBisCO complexes (Figure 8b). Even though the *C* value for 100:1 12 h Mg:RuBisCO increased by ∼64% compared to the value calculated for the 50:1, 12 h Mg:RuBisCO complexes, it was still not high enough to determine whether the knee of the adsorption isotherm is sharp and well-defined, suggesting that the knee of both isotherms cannot be used as a transitional point between the completion of the monolayer sorption and the beginning of the multilayer sorption.

Following the N_2_ gas adsorption screening, the 100:1 ratio 12 h Mg:RuBisCO complexes were subsequently used for evaluating CO_2_ adsorption at 25 °C (room temperature), 50 °C, and 75 °C, respectively. Analysis showed that the physisorption isotherm followed Type III adsorption with a Type H3 hysteresis loop (Figure 8c). All three adsorption isotherms showed that adsorption of CO_2_ began at a later pressure; for instance, adsorption did not occur until 100 mmHg (*P*/*P*_0_ = 0.132), 58 mmHg (*P*/*P*_0_ = 0.076), and 28.7 mmHg (*P*/*P*_0_ = 0.038) of CO_2_ were introduced when temperature varied from 25, 50, to 75 °C, respectively. In addition, a slow adsorption rate was observed at all 3 temperatures for *P*/*P*_0_ < 0.05. However, *P*/*P*_0_ > 0.5 showed a rapid rise of CO_2_ adsorption, specifically when testing at 25 °C, a much more drastic increase of CO_2_ adsorption happened with an increase of CO_2_ pressure around 415 mmHg (*P*/*P*_0_ = 0.54). Interestingly, upon increasing the temperature, the maximum adsorption capability of the samples decreased, confirming the exothermic nature of the adsorption process. For instance, the maximum adsorption at 25 °C was 1.9 cm^3^ g^–1^ (STP); meanwhile, at 50 and 75 °C it corresponded to 1.6 and 1.6 cm^3^ g^–1^ (STP), respectively, both showing decreases of 19% from the value recorded at 25 °C. Moreover, the CO_2_ desorption occurred at a slow linear rate and not until much lower pressures (less than ∼25 mmHg, *P*/*P*_0_ < 0.033) (Figure 8d).

## 4 Discussion

The formation of the metal–enzyme complexes at room temperature was systematically studied by using a combination of characterization techniques that allowed evaluation of such complexes' morphological, chemical, size, structural, and functional properties. Analysis revealed that synthesized complexes display morphological characteristics function of both the ratio and the time used. Poor structural organization was observed at lower ratios of metal to enzymes (i.e., 1.5:1 and 5:1, respectively) presumably due to the insufficient and/or weak coordination^168,169^ of the metal ions to the enzyme scaffolds. Previous analysis supports this hypothesis with research showing that metal ions can bridge functional groups of enzymes such as carboxylates,^170^ amines,^171^ and imidazoles.^172^ However, the limited availability of metal ions was shown to result in disordered morphologies.^173,174^

Spectral changes evaluated by FTIR supported that coordination will be a function of the ratio of metal used, with complexes formed at low ratio of metal to enzyme, showing that characteristic amide I (∼1650 cm^–1^) and amide II (∼1540 cm^–1^) bands of the enzyme in the complex remain largely unchanged when compared to those of the native enzyme. With an increase in ratio of metal, however, there was weakening of bands in the 500–600 cm^–1^ range, typically associated with metal–oxygen or metal–nitrogen coordination.^175,176^ The cyanide (−C≡N−) functional group^138,145^ between 2162 and 2037 cm^–1^ further confirmed the interaction between Mg ions and the enzyme, with previous analysis showing that such interactions could lead to σ-bonding–electrons transfer from its weakly antibonding σ-orbital to the metal– and π-bonding–electron acceptance from the metal through back-donation to its antibonding π-orbital.^177–179^ The amide I band observed from 1700 to 1649 cm^–1^ and known to be caused by the vibrations from carbonyl C=O, C–N stretch, and N–H bending of the amino acids bonds^141–144,146,147^ was shifted from the originally recorded 1650 cm^–1^, further suggesting perturbation of the carbonyl group due to the metal binding as well as possible coordination with aspartic and/or glutamic acid groups of the enzyme.^180^ Indeed, previous literature supported such a hypothesis with the protonation of the amine group on the aspartic acid ligand being shown to reduce charge to −1 due to the deprotonation of carboxylic acid moieties, thus requiring two ligands to achieve a charge balance with the Mg^2+^ center, as observed by Schmidbaur et al.^181,182^ as well as documented extensively by Case et al.^183^ In the case of glutamic acid, a bridging-bidentate configuration might have been the result through the influence of the pH environment where the binding between the metal and glutamic acid could have occurred via the α- and γ-carboxyl groups of the ligand, as observed via an ATR-FTIR study conducted by Parikh et al.^184^

Characteristic peaks for phosphoester bond were also presented as sharp peaks at 1234 cm^–1^ for C–OH with P=O,^148–150^ and at 1163, 1057, and 1016 cm^–1^ for aliphatic and aromatic C–O–C, C–O–P, C–O, and P–O^150,151^ groups, respectively. The peak at 883 cm^–1^ was attributed to the rocking vibration of the CH_2_,^148,152^ while the broad band from 850 to 550 cm^–1^ was presumably due to the stretching vibrations of Mg–O–Mg^153–156^ thus providing further evidence of coordination between the metal and functional groups on the enzyme scaffold. No significant peak broadenings in Mg:RuBisCO complexes across all time-points and ratio being analyzed were recorded, indicating that there are no major changes present in large enough abundance^185^ thus further confirming both SEM and EDX results. Large broadening of the peaks was previously associated with crystal imperfections (i.e., microstrains^185–188^ ) or with smaller crystallites being formed on the structures (typically <1 μm).^185,187,189–191^

The average hydrodynamic diameter of the complexes for either 50:1 and 100:1 generally remained within a relatively narrow range distribution (i.e., 0.5–7 μm), indicating that the complex formation was generally robust upon initiation. The size distribution presented as a function of both the metal-to-enzyme ratio and the reaction time used for synthesis, respectively, showed broadened distributions for the 50:1 relative to the 100:1 ratio, thus highlighting the kinetic and stoichiometry difference in the assembly process for these two ratios (Figure S6). Indeed, previous analysis has shown that elevated metal concentrations result in larger amounts of metal ions available to facilitate multiple coordination sites on enzyme scaffolds in solutions and thus to promote a greater number of nucleation centers.^192,193^

This is supported by previous classical nucleation theory (CNT)^194–197^ studies that showed that the nucleation rate and subsequent complex size distribution depend strongly on the concentration of metal ions present in solution as well as the reaction time, with regular sizes and narrower distribution expected for higher metal–enzyme ratios. A peak in the energy barrier also showed how the critical size of a crystal formation was dictated.^198,199^ Thus, our results support the theory that with limited nucleation sites particles tend to grow irregularly as the available metal ions coordinate less uniformly. The slight increase in size recorded for the 100:1 relative to the 50:1 ratio, especially at longer synthesis times, may be attributed to continued growth and/or surface rearrangement of the metal ions, allowing for more complete coordination and stabilization of the complexes as supported by Vekilov et al. studies for lysozyme protein crystallization.^200,201^

The observed enhanced nucleation at higher metal loadings was also supported by XRD data, which recorded sharper and more defined peaks, hinting at improved crystallinity of the synthesized complexes. In particular, cross-comparison between the time points and synthesis ratios as shown in Figure 9 showed the higher intensity peaks being shifted to lower 2θ angles to support the increase in ionic radii and the amount of ions being incorporated in the crystal with ratio and time variations,^202–204^ respectively. The larger shift presented in the 50:1 Mg:RuBisCO synthesis also supported the nucleation and growth phase of the crystals at this ratio, complementing the size analysis. A similar trend was noticed in a study about nucleation time for the formation of ZnO nanoparticles, with analysis showing that a larger number of crystals would grow and would reduce the local reactants concentration if the nucleation proceeded rapidly, thus leading to defects appearing into and onto the crystal and affecting the crystal quality.^204,205^ Complementarily, the reduced shifts in the 100:1 ratio indicated an overall more stable, toward thermodynamic equilibrium crystal growth process,^206^ again supporting the size analysis and narrower distribution differences observed between the different time of synthesis.

**Figure 9:**
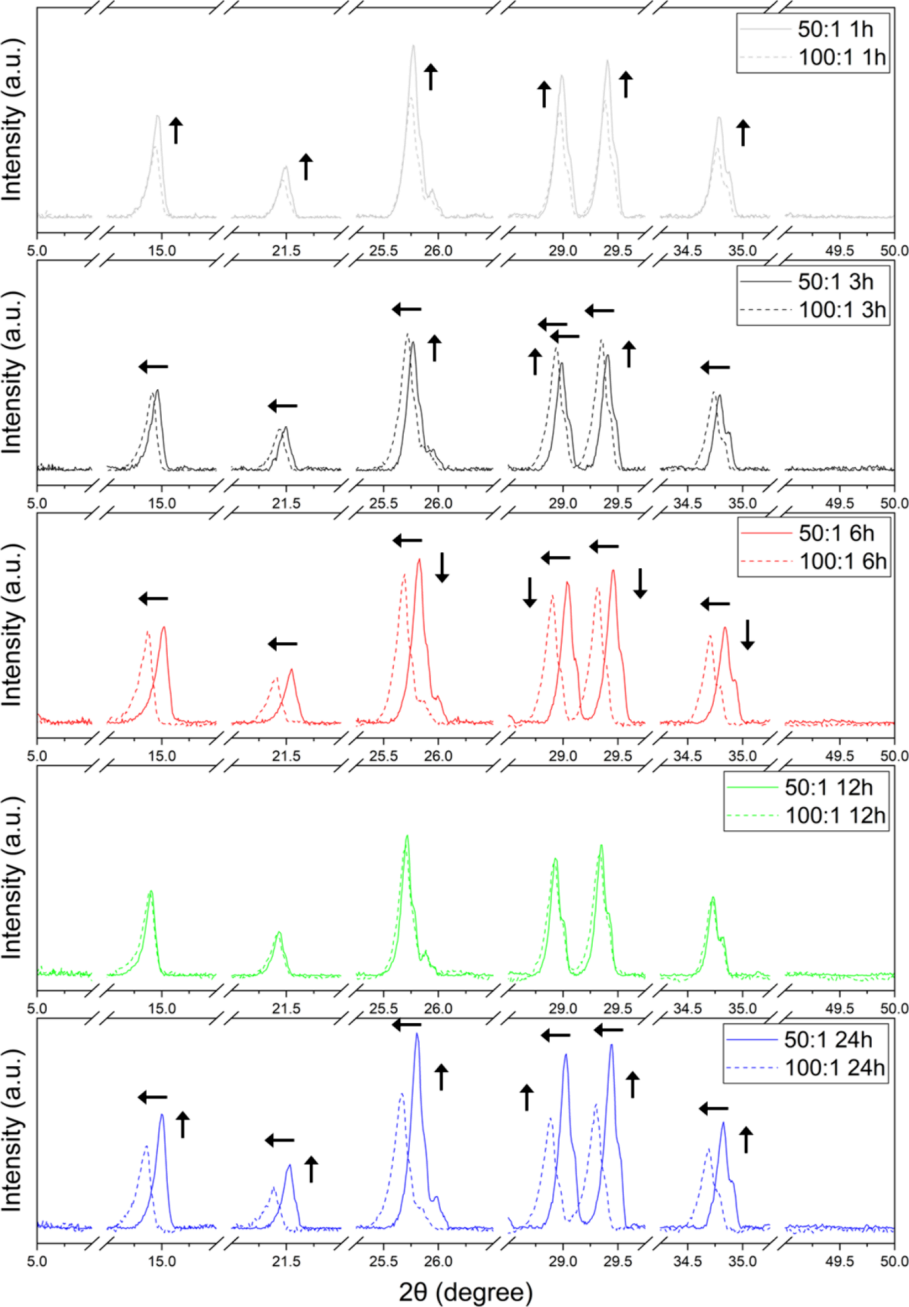
Cross-comparisons of all time points of synthesis for the two ratios being used, namely, 50:1 and 100:1 ratios of Mg:RuBisCO. Arrows indicate the shifts in peaks, either in intensity or localization.

In addition, previous reports showed that peak positions will remain the same, but their relative intensities could change if certain microcrystals were growing in certain directions, were of certain shapes (i.e., changes from granular to needle or plate for instance), and/or the surface area of each diffraction face, respectively.^207,208^ These reports support our SEM analysis which showed a mixture of complexes’ orientations; in particular, as the complexes’ shapes were nonspherical, upon their drying for either XRD or SEM analysis, they could have oriented in nonrandom directions.^189,209^ For example, a sample of cube-shaped particles dried or precipitated from solutions was shown to orient with their flat faces parallel to the drying surface.^189,210,211^ The XRD pattern with sharp, dominant peaks (i.e., (110), (102), (122), (131), (311), and (104), (122), (131), (311)) as shown by intensity analysis relative to the MgCl_2_ control, is consistent with particles having much larger crystalline domain sizes in those domains more likely due to preferential orientation and binding of the Mg ions.^207,208^ It was much less likely that rhomboidric cubes would dry with their corners or edges touching the drying surface, and therefore, the powder of such structures is expected to be preferentially oriented in the crystallographic direction corresponding to those faces. Previous studies indeed supported that structures may exhibit preferred orientation depending on the size of the various shapes.^189,212^

A score of 63 with 0.032° 2θ displacement calculated by the proprietary HighScore software for the Mg:RuBisCO supported the hypothesis that synthesized complexes are newberyite structures which were previously found in magnesium phosphate-based crystals.^213^ Such formation of newberyite was shown to be influenced by the concentrations of Mg^2+^ ions^214–217^ with higher number of ions leading to the formation of more of such structures and with Mg coordination resembling Mg-binding sites in proteins as Mg^2+^ coordinates with oxygen donors from side chains or backbone carbonyls^213,218^ to stabilize an enzyme conformation and promote crystallinity.

The gas adsorption results indicate a BET surface area of 1.5–2.4 m^2^/g (with the addition of total pore volume for the complex being ∼0.5 cm^3^/g). This area is significantly lower than that of the pristine metal–organic framework (MOF) for instance, with studies showing that zeolitic imidazolate frameworks (ZIFs) present their surface areas ranging from 1000 to 2000 m^2^/g^219,220^ and MMSs that have surface areas ranging from 800 to 1200 m^2^/g.^221–223^

Even though much lower, the complexes formed in this research demonstrate the formation of an accessible surface area for gas adsorption, with the recorded BET being slightly higher than that of the typical globular macromolecules like enzymes known to be <1.5 m^2^/g, with literature reporting BET surface areas of IgG monoclonal antibodies (mAb) at 0.6–1.4 m^2^/g,^224,225^ natural cellulose fibers at 0.5 m^2^/g,^226^ bovine serum albumin (BSA) at 0.6–2.2 m^2^/g,^227^ lactose at 0.29 m^2^/g,^228^ and sucrose at 0.62–1.21 m^2^/g for instance.^229^ At the time of this report, there were no reports on the specific surface area of CA or FDH enzymes, which are known prominent candidates for materials formation to be used in CO_2_ adsorption studies. Moreover, when combining the measured surface area with the pore volume which was relatively high suggesting meso- or microporosity, analysis of the approach presented herein demonstrates added functionality of the synthesized complex for synthetic applications.

When the complex was evaluated for CO_2_ adsorption at 25, 50, and 75 °C, respectively, it was found to exhibit close to 2 cm^3^/g adsorption capability, with a clear decreasing trend as the temperature increased. The temperature dependence confirms that CO_2_ adsorption is exothermic in nature and governed by weak, physical interactions like weak van der Waals forces.^230,231^ Indeed, our Supporting Information analysis based on CO_2_ chemisorption testing and in situ KBr FTIR (presented in Figure S7 and Figure S8, respectively) confirmed that the CO_2_ binding mechanism to the 100:1 12 h synthesized complex was only observable through physical adsorption. For the CO_2_ chemisorption adsorption isotherm study, to observe any active sites or a covalent bonding between the adsorbate and the adsorbent, the adsorption typically needs to decrease in repeated runs/exposures to the adsorbate which was not observed in this study.^232,233^

The low gas uptake is not unexpected given the lack of accessible surface area and the absence of strong CO_2_-philic functional groups (e.g., amines or open metal sites) on the surface of the complex. It is thus proposed that to enhance CO_2_ uptake in such metal–enzyme complexes, amine functionalization could be employed to significantly increase the level of CO_2_ capture, even if the overall surface area might remain low. Indeed, research in our group and others has previously shown that enzymes offer scaffolds for cross-linking amine residues through bifunctional reagents like glutaraldehyde or EDC/NHS chemistry.^234,235^ Moreover, it was found that adding amino groups to porous materials greatly improves their capacity to adsorb CO_2_ molecules due to the favorable Lewis acid–base interactions created.^236–238^ A detailed mechanism of CO_2_ reaction to primary and secondary amines was thoroughly discussed by Zelenak et al.,^236^ Muchan et al.,^238^ and Rinker et al.,^239^ just to name a few.

Although the observed CO_2_ uptake was reduced (i.e., close to 2 cm^3^/g), the recorded trends are consistent with the ones recorded for other biocomposites designed for localized capture of gases, rather than for gas bulk storage.^240–242^ In particular, Hou et al. have shown that biological-based materials like quaternized bamboo cellulose hold significant potential for direct air capture due to their moisture-induced difference in the adsorption performance.^242^ As such, this work potentially opens new directions for biocompatible, low-energy, CO_2_ adsorption technologies based on enzymes. Through such a proof of principle departed from traditional work focused on enzymes in conjunction with porous materials and evaluated for CO_2_ adsorbing capabilities like the CA enzymes used with Zn^2+^-based porous materials^72,243,244^ or the FDH enzymes on porous materials like ZIF-8,^245^ our work further benefits from the unique integration of Mg^2+^, which has lower electronegativity (Pauling scale of 1.31 for magnesium compared to 1.91 and 1.65 of nickel and zinc, respectively^246^), thus expecting to reduce the Lewis basicity to results in better effectiveness in CO_2_ hydration if transformation of the absorbed gases is to be sought in future applications.^247^

## 5 Conclusions

In this work, a metal–enzyme complex was successfully synthesized via a mild room temperature strategy. The synthesis approach enabled the formation of complexes with morphologies, sizes, and crystallinities optimized based on the ratio of metal to enzyme and time used for synthesis, respectively. Preliminary gas adsorption studies demonstrated N_2_ and CO_2_ uptake in the synthesized complexes, with a characteristic decrease in adsorption at elevated temperatures, indicative of physisorption-driven interactions for the gas. This research represents a proof of principle concept for the design of hybrid materials where CO_2_ adsorption can facilitate downstream transformation under mild conditions to expand material’s applicability in carbon capture technologies.

## Supplementary Material



## Notes

SEM image (Figure S1); elemental composition (Table S1 and Figure S2); ATR-FTIR (Figure S3); diffraction data (Table S2); IUPAC’s isotherm and hysteresis classification as well as pore sizes (Figure S4): Langmuir surface areas and adsorption capacities (Table S3); pore size distributions (Figure S5); particle size analyses (Figure S6); CO_2_ chemisorption adsorptions (Figure S7); in situ KBr FTIR data (Figure S8) (PDF)
