# Supplementary material for: Synthesis and Characterization of Rubisco–Magnesium Complexes for Potential Gas Adsorption Applications
Source: ACS Omega. 2026 Jan 8;11(3):4062–84. doi: 10.1021/acsomega.5c08077 (PMC12854619; doi:10.1021/acsomega.5c08077)
Supplement: Supplementary file 1 [file ao5c08077_si_001.pdf]

Supporting Information

Synthesis and Characterization of Rubisco–Magnesium Complexes for Potential Gas Adsorption Applications

AUTHORS: *Gia Huy Pham †, Elizabeth Willenborg †, Emily Weber †, Brandon Robinson †, Cerasela Zoica Dinu *,†*

AUTHOR ADDRESS: † Department of Chemical and Biomedical Engineering, West Virginia University, Morgantown, West Virginia, 26506, United States

KEYWORDS: enzyme, metal conjugation, time- and dose-dependent, hybrid structure, CO_2 binding, Magnesium-RuBisCO complex, enzymatic gas sensing application, RuBisCO

, West Virginia University, Morgantown, West Virginia, 26506, United States

* Corresponding Author: Cerasela-Zoica.Dinu@mail.wvu.edu

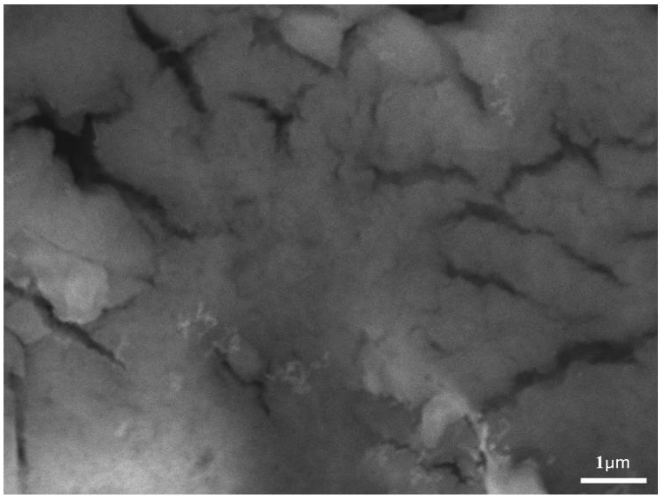

Figure S1. SEM image of $MgCl_2 \cdot 6H_2O$ at 12,000x with a 1 μm scale bar shown at the bottom right corner of the image.

Table S1. Elemental Composition with average and standard deviation included for each synthesis conditions (left to right- 1, 3, 6, 12, and 24 h with respect to mass ratio, top-down 5:1, 50:1, and 100:1 respectively).

**Oxygen (%)**	**1 h**	**3 h**	**6 h**	**12 h**	**24 h**
**5:1**	49.79 (± 6.83)	54.73 (± 3.46)	53.98 (± 1.12)	52.29 (± 3.85)	39.13 (± 13.41)
**50:1**	45.71 (± 4.63)	53.95 (± 2.30)	55.66 (± 1.71)	48.08 (± 11.54)	50.80 (± 5.63)
**100:1**	46.84 (± 4.22)	53.15 (± 6.60)	55.57 (± 1.78)	53.79 (± 3.68)	47.97 (± 7.08)
**Magnesium**	**1 h**	**3 h**	**6 h**	**12 h**	**24 h**
**5:1**	16.11 (± 2.74)	17.76 (± 1.32)	16.01 (± 1.85)	17.80 (± 1.18)	15.02 (± 4.42)
**50:1**	18.78 (± 0.30)	17.35 (± 0.30)	17.47 (± 0.54)	17.44 (± 3.58)	16.62 (± 1.23)
**100:1**	19.91 (± 0.83)	18.25 (± 0.43)	17.26 (± 1.70)	18.19 (± 1.35)	17.08 (± 1.29)
**Phosphorus**	**1 h**	**3 h**	**6 h**	**12 h**	**24 h**
**5:1**	24.72 (± 5.65)	21.26 (± 4.75)	19.98 (± 3.55)	24.60 (± 4.60)	39.57 (± 14.75)
**50:1**	28.55 (± 4.79)	20.95 (± 2.46)	21.65 (± 0.76)	30.11 (± 12.84)	23.80 (± 3.28)
**100:1**	27.66 (± 4.13)	24.92 (± 5.53)	20.95 (± 2.43)	24.18 (± 3.85)	29.67 (± 8.67)
**Carbon**	**1 h**	**3 h**	**6 h**	**12 h**	**24 h**
**5:1**	2.78 (± 2.00)	1.41 (± 0.64)	10.24 (± 5.72)	4.19 (± 2.55)	4.14 (± 1.39)
**50:1**	4.94 (± 0.22)	5.02 (± 0.71)	3.94 (± 1.86)	2.48 (± 2.16)	4.76 (± 4.71)
**100:1**	2.70 (± 0.88)	2.87 (± 1.11)	6.40 (± 6.83)	3.24 (± 2.39)	4.20 (± 5.14)

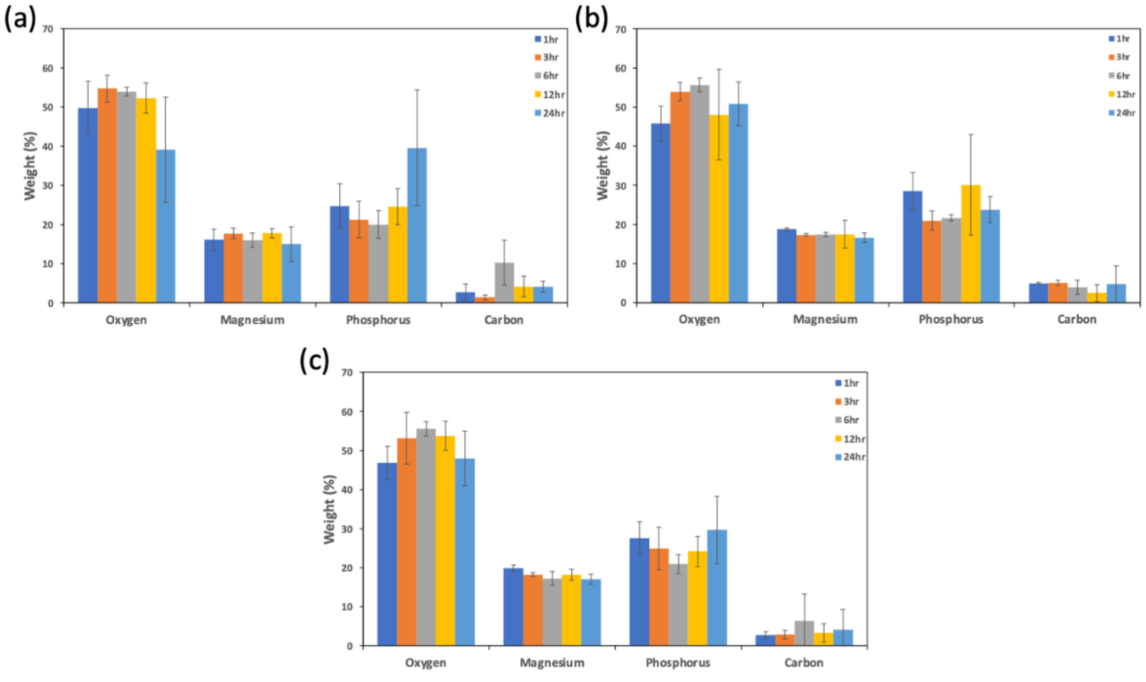

Figure S2. Elemental composition of Mg:RuBisCO with different mass ratios: (a) 5:1, (b) 50:1, and (c) 100:1. Dark blue represents observations for 1h synthesis, while orange, gray, yellow and light blue represents analysis for 3, 6, 12 and 24 h synthesis respectively.

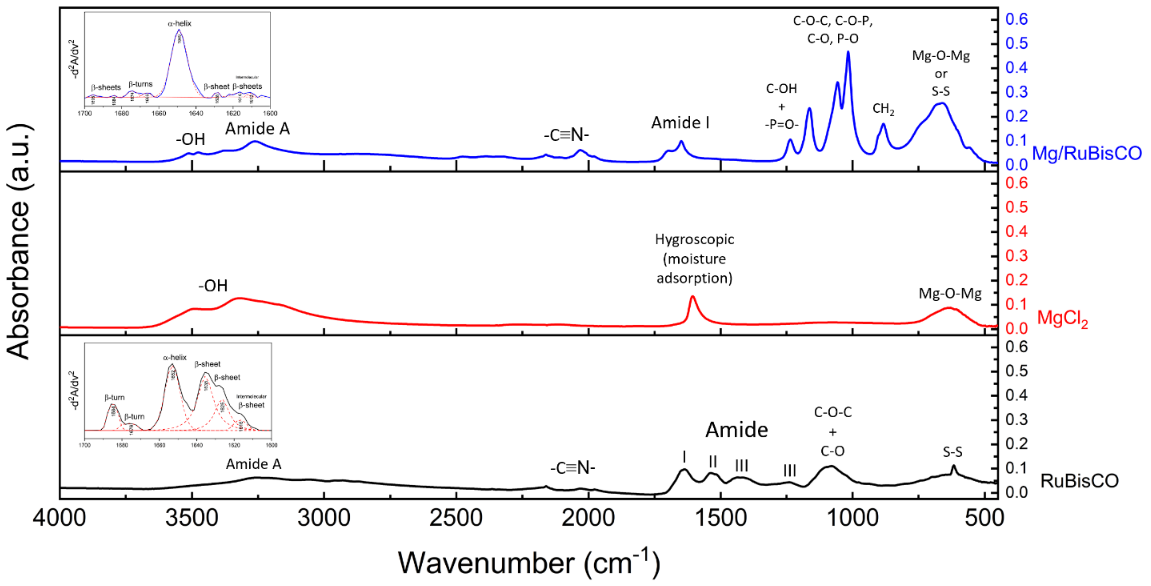

Figure S3. ATR-FTIR of (top) 100:1 Mg:RuBisCO with (inset) its secondary protein structure analyzed from the Amide I band, (middle) $MgCl_2{\cdot}6H_2O$, and (bottom) RuBisCO with (inset) its secondary protein structure analyzed from the Amide I band.

Table S2. Diffraction data of $MgCl_2 \cdot 6H_2O$, including peak index based on the 2θ location, the d-spacing (Å), the relative intensity, and the (hkl) indices of each peak.

**2θ (°)**	**d-spacing (Å)**	**Relative Intensity (%)**	**Indices (hkl)**
15.446	5.732	16.76	(110)
21.745	4.094	100.00	(111)
25.114	3.543	22.52	(020)
30.047	2.972	34.45	(310)
31.089	2.874	55.94	(220)
32.738	2.733	15.04	($\bar{3}$11)
32.897	2.720	43.15	($\bar{1}$12)
33.975	2.637	49.28	($\bar{2}$21)
35.037	2.559	5.02	(221)
39.095	2.302	23.47	(022)
40.496	2.225	27.71	(401)
41.767	2.161	2.96	($\bar{1}$31)
42.217	2.139	5.55	(131)
43.899	2.061	7.19	(312)
44.228	2.046	12.4	(222)

(a)

(b)

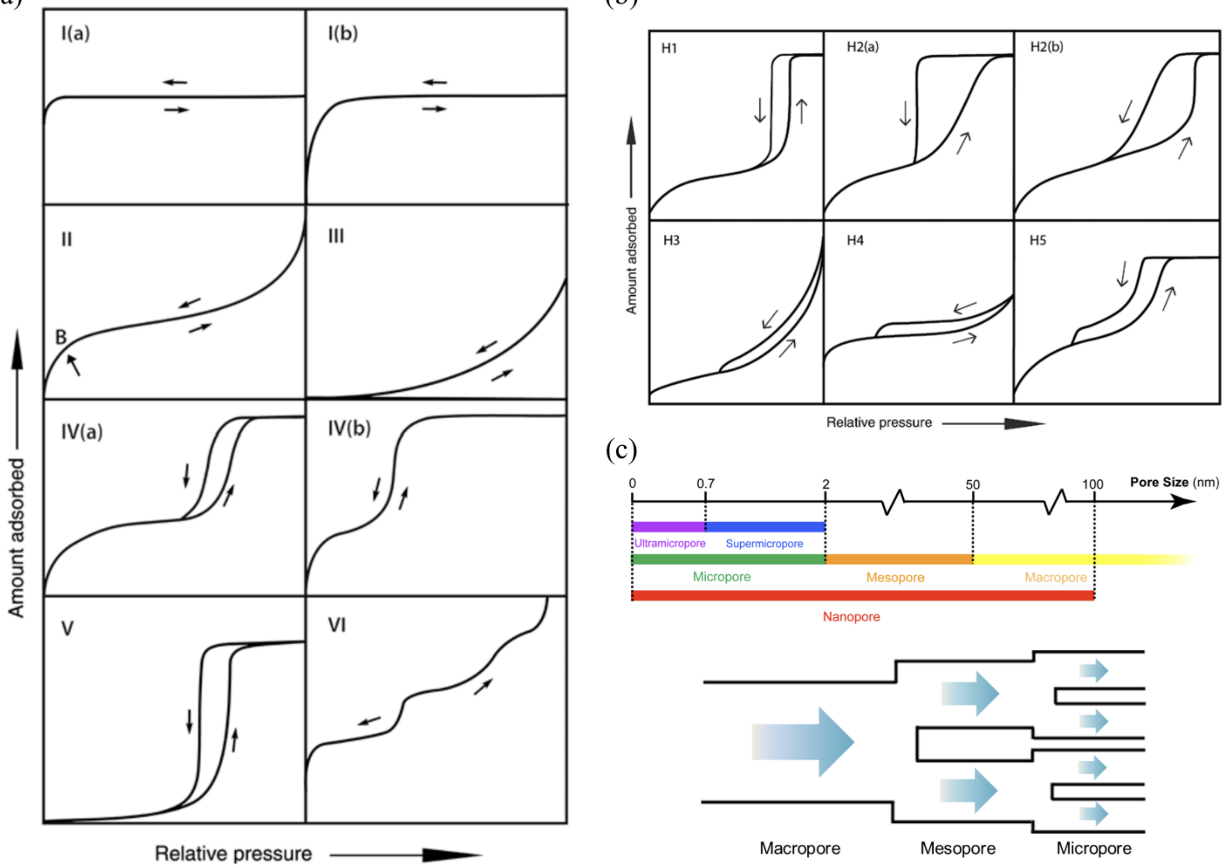

Figure S4. IUPAC's classification for the (a) physisorption isotherm [1], (b) hysteresis loop [1] with copyright from ©2015 IUPAC & De Gruyter; (c) pores sizes based on pore width with a schematic ion diffusion pattern in a porous structure [2].

Table S3. $S_{Langmuir}$ (m^2/g), b, Q_m (cm^3/g STP), slope (g/cm^3 STP), y-intercept (g/cm^3 STP) calculations based on the Langmuir model for 50:1 and 100:1 12h Mg/RuBisCO samples (n=3).

Ratio/Time Mg/RuBisCO	$S_{Langmuir}$ (m^2/g)	b ($mmHg^{-1}$)	$Q_{m,Langmuir}$ (cm^3/g STP)
50:1 12h	2.841 ± 0.208	0.012190 ± 0.00243	0.6527 ± 0.0364
100:1 12h	3.6974 ± 0.135	0.029871 ± 0.00471	0.8495 ± 0.00227

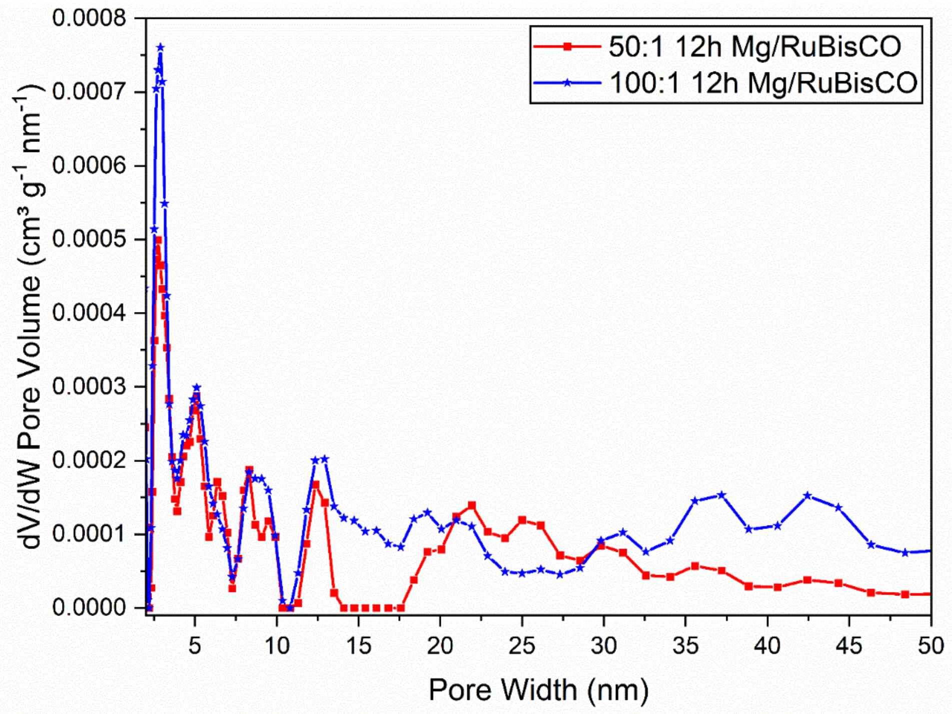

Figure S5. The pore size distribution in the mesopore region (width = 2-50nm) calculated by NL-DFT from the N_2 adsorption isotherm for a respective (red, square) 50:1 12h Mg:RuBisCO and a respective (blue, star) 100:1 12h Mg:RuBisCO sample.

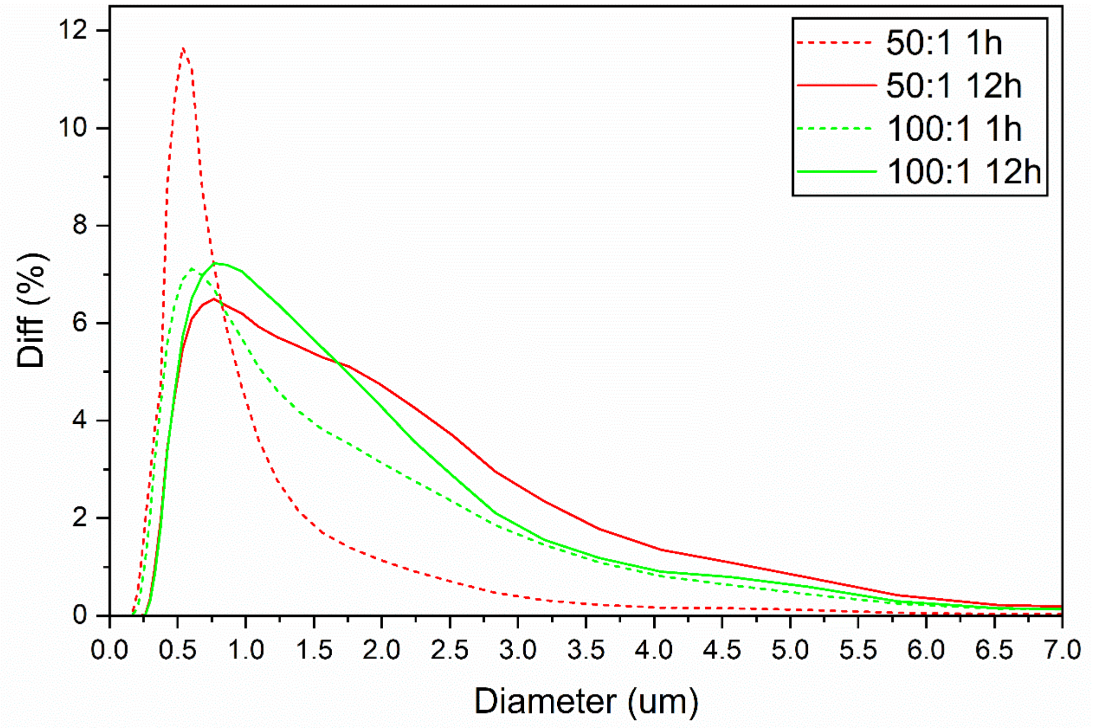

Figure S6. Particle size analysis of 50:1 (red) and 100:1 (green) Mg:RuBisCO between 1h (dash) and 12h (solid).

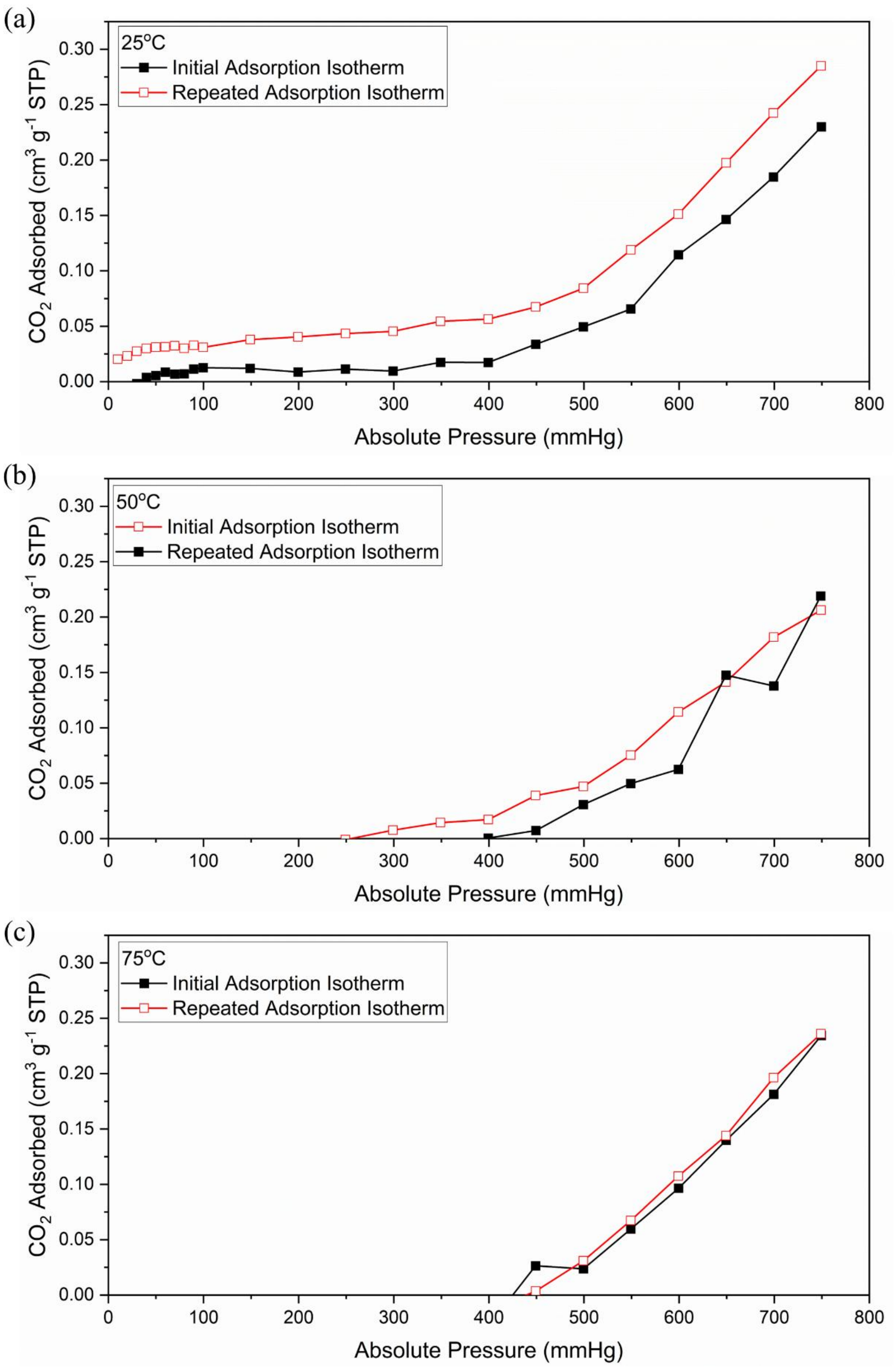

Figure S7. CO_2 chemisorption adsorption at (a) 25°C, (b) 50°C, and (c) 75°C.

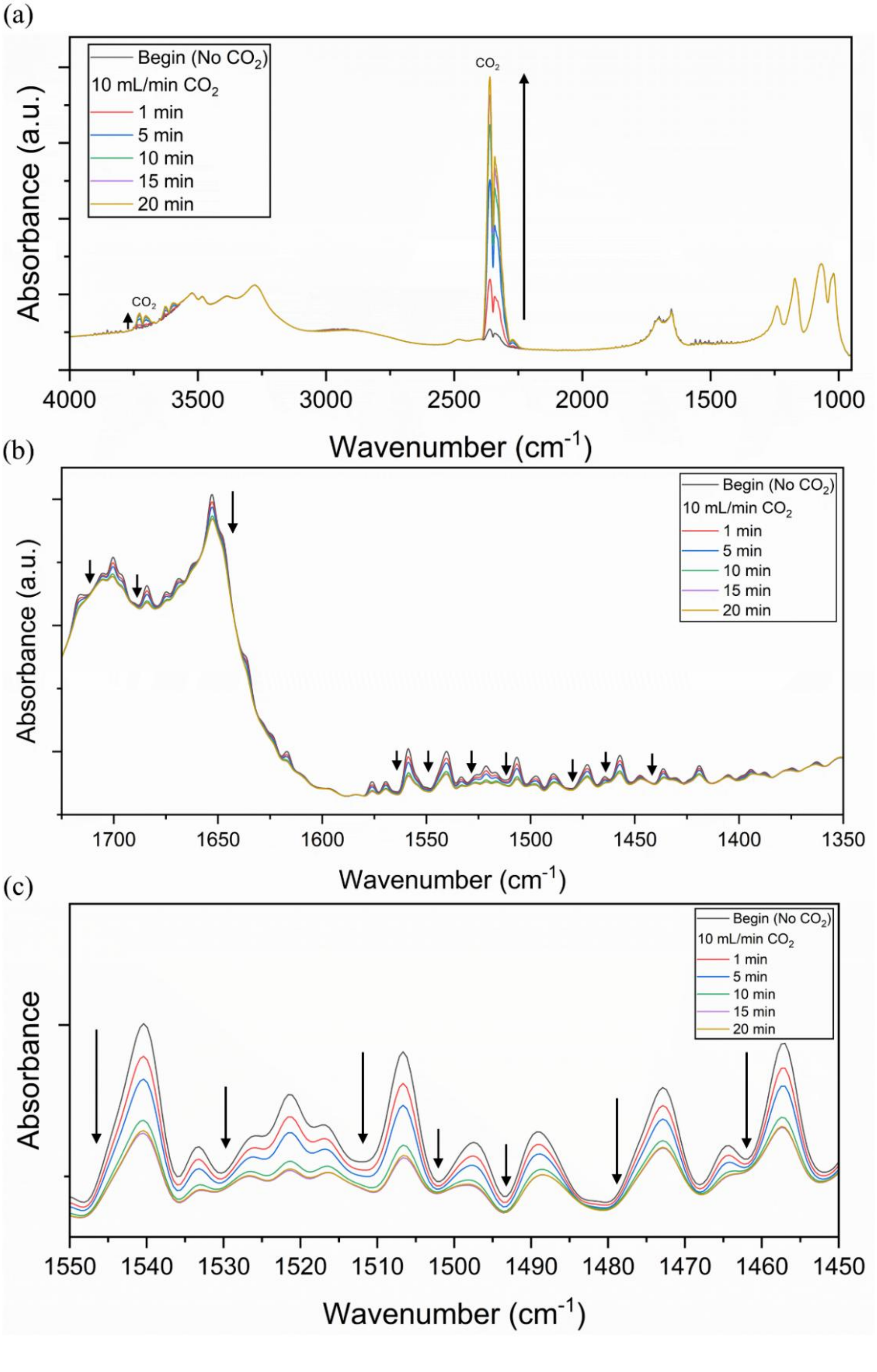

Figure S8. In situ KBr FTIR of a representative 100:1 12h Mg:RuBisCO sample showing the (a) full spectra, (b) bicarbonate/carbonate region from 1350 cm^{-1} to 1750 cm^{-1}, and (c) primary amine region from 1450 cm^{-1} to 1550 cm^{-1}.

References:

(1) Thommes, M.; Kaneko, K.; Neimark, A. V.; Olivier, J. P.; Rodriguez-Reinoso, F.; Rouquerol, J.; Sing, K. S. W. Physisorption of gases, with special reference to the evaluation of surface area and pore size distribution (IUPAC Technical Report). **2015**, *87* (9-10), 1051-1069.

(2) Liu, T.; Zhang, F.; Song, Y.; Li, Y. Revitalizing carbon supercapacitor electrodes with hierarchical porous structures. *Journal of Materials Chemistry A* **2017**, *5* (34), 17705-17733.
